# Identifying tandem Ankyrin repeats in protein structures

**DOI:** 10.1186/s12859-014-0440-9

**Published:** 2014-12-30

**Authors:** Broto Chakrabarty, Nita Parekh

**Affiliations:** Centre for Computational Natural Sciences and Bioinformatics, International Institute of Information Technology, Hyderabad, India

**Keywords:** Ankyrin repeat, Protein contact network, Graph theory

## Abstract

**Background:**

Tandem repetition of structural motifs in proteins is frequently observed across all forms of life. Topology of repeating unit and its frequency of occurrence are associated to a wide range of structural and functional roles in diverse proteins, and defects in repeat proteins have been associated with a number of diseases. It is thus desirable to accurately identify specific repeat type and its copy number. Weak evolutionary constraints on repeat units and insertions/deletions between them make their identification difficult at the sequence level and structure based approaches are desired. The proposed graph spectral approach is based on protein structure represented as a graph for detecting one of the most frequently observed structural repeats, Ankyrin repeat.

**Results:**

It has been shown in a large number of studies that 3-dimensional topology of a protein structure is well captured by a graph, making it possible to analyze a complex protein structure as a mathematical entity. In this study we show that eigen spectra profile of a protein structure graph exhibits a unique repetitive profile for contiguous repeating units enabling the detection of the repeat region and the repeat type. The proposed approach uses a non-redundant set of 58 Ankyrin proteins to define rules for the detection of Ankyrin repeat motifs. It is evaluated on a set of 370 proteins comprising 125 known Ankyrin proteins and remaining non-solenoid proteins and the prediction compared with UniProt annotation, sequence-based approach, RADAR, and structure-based approach, ConSole. To show the efficacy of the approach, we analyzed the complete PDB structural database and identified 641 previously unrecognized Ankyrin repeat proteins. We observe a unique eigen spectra profile for different repeat types and show that the method can be easily extended to detect other repeat types. It is implemented as a web server, AnkPred. It is freely available at *‘bioinf.iiit.ac.in/AnkPred’*.

**Conclusions:**

AnkPred provides an elegant and computationally efficient graph-based approach for detecting Ankyrin structural repeats in proteins. By analyzing the eigen spectra of the protein structure graph and secondary structure information, characteristic features of a known repeat family are identified. This method is especially useful in correctly identifying new members of a repeat family.

**Electronic supplementary material:**

The online version of this article (doi:10.1186/s12859-014-0440-9) contains supplementary material, which is available to authorized users.

## Background

In this study we address an important pattern recognition problem in protein structures, *viz*. the identification of structural tandem repeats. Repeats are ubiquitous in protein sequences and vary considerably from single amino acid repetitions, e.g., runs of glutamine in the protein huntingtin, to large globular domains of size 100 or more residues that fold independently [1]. The structural classification of repetitive proteins based on the repeat lengths and the possible 3-dimensional structure of these proteins has been given by Kajava (2001) [2]. Repeats of intermediate length of 20–50 amino acids are most commonly observed in proteins to form integrated assemblies providing multiple binding sites. This class of non-globular repeat proteins form various 3-dimensional folds, *viz.*, spirals, solenoids, closed structures, etc. [3]. Examples of such repeats include leucine-rich repeat (LRR), Ankyrin repeat (ANK), armadillo (ARM) /HEAT repeat, tetratricopeptide repeat (TPRs), Kelch repeat, etc.

Earlier approaches for the identification of repeats in protein sequences range from methods based on Fourier analysis of amino acid sequences [4-7], to short-string searches [8,9], sequence-alignment based approaches [10-13], and HMM-profile based methods [14,15]. The Fourier transform methods fail in the presence of insertions between the repeating units, while the performance of the alignment-based methods fail when there is weak, non-detectable similarity between the repeating units. A comprehensive review of the sequence-based approaches for the detection of protein repeats is given by Kajava (2012) [3]. As each copy of a repeat accumulates independent uncoordinated mutations over the evolution, weak similarities between repeated copies make their identification difficult and in certain cases non-detectable by the sequence-based approaches. The low similarities imply that the functional constraints on individual repeats are relatively weak when compared to the constraints imposed on the repeat assembly as a whole. Since proteins are more conserved at the structure level, their identification at the structural level is desirable. Over the last decade various structure based methods have been proposed to predict internal repeats in protein structures. The earlier approaches, DAVROS [16] and OPASS [17], based on structure-structure alignment of protein to itself, are computationally very intensive. Both these algorithms are no longer maintained, nor is the Propeat Database [18] constructed using OPASS algorithm. Swelfe [19] and ProSTRIP [20] methods are based on the self-alignment of the sequence of α characters (the backbone dihedral angles) using dynamic programming for detecting internal structural repeats. These methods, based on the periodicity of dihedral angles (as a result of repetition of secondary structure elements), fail in case of large insertions/deletions. The Internal Repeat Identification System (IRIS) performs both sequence-based and structure-based approach for identifying internal repeats. When structural information for a protein is available, it verifies the sequence-based prediction by structure-comparison within itself, or with their benchmark database of internal repeat units [21]. Another recent method based on the distribution of the structural alignment of continuous fragments is given by Parra et al. [22]. RAPHAEL, based on Fourier analysis of C_α_ coordinates in combination with support vector machine (SVM), tries to mimic visual interpretation of a manual expert and classifies a protein into solenoid/non-solenoid class [23]. A large number of novel solenoid repeat proteins have been identified by this approach. With a large number of protein structures now available, reliable automatic methods of analyses are required. Algorithms from computer science have been widely used for identifying biological patterns and concepts from graph theory have been promising. ConSole [24] is one such recent method which transforms the protein structure into a network and implements a rule based machine learning technique to identify solenoid repeats in proteins. Its performance on benchmark datasets is shown by the authors to be better than RAPHAEL. Here we propose a computationally efficient algorithm for the identification of an important class of protein repeat family, *viz*., Ankyrin (ANK) repeats, based on graph theory and secondary structure architecture of the repeating unit. The proposed approach is observed to perform better compared to ConSole especially in identifying the terminal repeats on the dataset of known ANK repeat proteins. The complete Protein Data Bank (PDB) structures are also analyzed to identify previously unrecognized ANK repeat proteins.

The particular architecture of proteins containing repeats make them attractive targets for protein engineering, being involved in innumerable biological processes as binding molecules, *viz*., cell-cycle regulation, transcriptional regulation, cell differentiation, apoptosis, plant defence or bacterial invasion [25]. The design and engineering of repeat proteins may help to elucidate their structural and biophysical properties, such as the dependence of stability and folding on the number of repeats, as well as the importance of key intra- and inter-repeat interactions and hence considerable effort is being made in this direction. This can have important biotechnological or medical applications. So far, consensus based on multiple sequence alignment of homologous proteins has been used for the design of repeat proteins. However, because of low sequence similarity among repeating motifs, and their occurrence in non-homologous proteins, identifying repeats at the structural level and building the consensus on structure-based multiple alignment of repeating units will be more reliable. And identification of repeats at the structural level forms the first step in this direction.

The design and engineering of a number of repeat proteins has been carried out, e.g., ANK (called DARPins), TPR and LRR. In this paper we present in detail the training and performance of the proposed approach on ANK repeat proteins and then show that it can be extended for the detection of other repeats proteins, TPR, LRR, ARM/HEAT and Kelch. Below we briefly discuss the feature and function of ANK and other repeats.

### Ankyrin repeat

**Figure 1:**
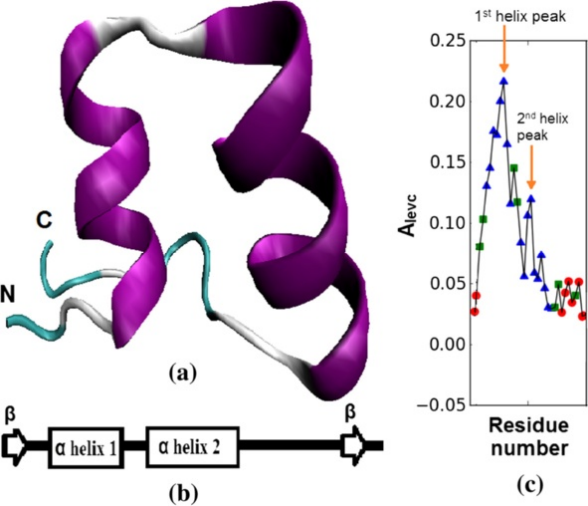
**Ankyrin repeat motif. (a)** The second copy of the ANK structural motif in designed protein 1N0R. **(b)** Schematic diagram showing the secondary structure arrangement in the ANK motif. **(c)** The principal eigenvector of adjacency matrix plotted for the ANK motif in **(a)**.

The Ankyrin repeat (ANK) is one of the most frequently observed structural motifs in proteins, especially in eukaryotes. It was first discovered in signalling proteins in yeast cell cycle regulator Cdc10 containing 24 copies of this repeat. In general, 4 to 6 copies of the repeat stack onto each other to form an elongated structure with a continuous hydrophobic core and a large solvent-accessible surface [26]. The protein-protein interaction module is involved in a diverse set of cellular functions, such as transcriptional initiators, cell-cycle regulators, cytoskeletal, ion transporters and signal transducers, and consequently, defects in Ankyrin repeat proteins have been associated with a number of human diseases [27]. For example, mutation in the ANK1 gene producing the erythrocyte ankyrin protein may lead to hereditary spherocytosis [28]. Each repeat typically consists of 30–34 amino acid residues comprising two anti-parallel α-helices and a long loop ending in a β-hairpin (shown in Figure 1(a)) and schematically in Figure 1(b), forming a scaffold for specific, high-affinity molecular interactions. In contrast to many other protein-protein binding motifs, it has been observed that the Ankyrin repeat motif is better characterized by its tertiary structure rather than by a specific function, because of the pronounced sequence variation in the individual repeats and in the copy number variation across various protein families.

### Other repeats

The analysis of the proposed graph based approach is also discussed for other repeat types, such as Tetratricopeptide repeat (TPR), Armadillo (ARM), Leucine-rich repeat (LRR) and Kelch. The example repeats have been chosen to represent different structural classes α, β and α/β, with some, such as TPR very similar to the ANK motif, while LRR and Kelch that have very different repeat lengths and architecture compared to ANK motif. A TPR motif is 34 residues long α-domain motif with two anti-parallel α helices forming the helix-turn-helix core of the repeat and the contiguous repeating units stack to form a super-helical structure [29]. ARM is another example of α-domain repeat, typically 42 residues with each repeat unit comprising three helices and the contiguous repeats form super-helical structure [30]. Kelch repeat forms a β propeller structure with 44–56 residues constituting 4–6 anti-parallel β strands in each repeat unit [31]. A typical LRR repeat unit with an anti-parallel helix-strand motif 24 residues long is an example of α/β domain motif and contiguous copies form a horse-shoe like structure [32]. Typically a repeat protein contains 4–8 copies of repeat, but higher copies are also observed depending on the function of the protein. The arrangement of repeating units in each of the repeat types provides structural stability and forms binding pockets for a wide range of protein-protein interactions.

## Methods

**Figure 2:**
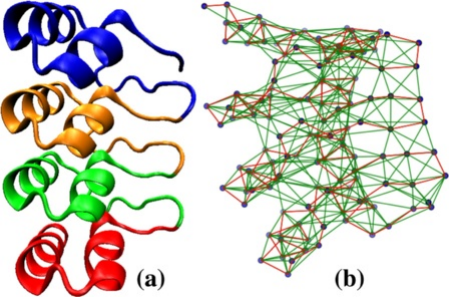
**Designed protein 1N0R (chain A).** **(a)** 3-D structure, and **(b)** protein contact network.

To represent the 3-dimensional protein structure as a graph, we consider the backbone *C*_α_ carbon atoms as nodes and draw edges between them if they are within a cut-off distance, *R*_*c*_ (~7 Å) [33]. The choice of *R*_*c*_ depends on the kind of interactions considered and for graphs constructed using *C*_α_, the value of *R*_*c*_ is typically chosen to be around two times the peptide bond-length to include all possible non-covalent interactions that are known to play a significant role in protein folding. The graph thus constructed is commonly referred to as a *protein contact network* (PCN) or *protein structure graph* (PSG) in literature and the connectivity information of *n* nodes (residues) is represented by a symmetric *n × n* adjacency matrix of the graph, whose elements, *A*_*ij*_, are given by:

$$ {A}_{ij}=1,\  if\ {d}_{ij}\le {R}_c\  and\ i\ne j $$

$$ {A}_{ij}=0,\  if\ {d}_{ij}>{R}_c\  or\ i=j $$

where ${d}_{ij}=\sqrt{{\left({x}_i-{x}_j\right)}^2+{\left({y}_i-{y}_j\right)}^2+{\left({z}_i-{z}_j\right)}^2}$ is the Euclidean distance between (*i, j*) pair, (*x*_*i*_*, y*_*i*_*, z*_*i*_) the coordinates of the *i*^*th*^*C*_α_ carbon atom, and *i, j* = 1, 2 … *n*, *n* being the number of *C*_α_ atoms (nodes) in the graph. Nodes joined by an edge are called *adjacent*, and the degree of the *i*^*th*^ node is defined as the number of its adjacent nodes: ${k}_i={\displaystyle \sum_j}{A}_{ij}$. It may be noticed that the 3-dimensional topology of the designed Ankyrin protein, 1N0R (Figure 2(a)) is captured very well by the protein contact network constructed using *C*_α_ atoms as nodes in Figure 2(b).

The degree gives the connectivity information of each node in the graph but all connections are not equal in the sense that, nodes connected to highly connected nodes contribute more compared to those connected to ones with fewer connections. This information is captured in the eigen spectra of the adjacency matrix. The principal eigen vector components (corresponding to the largest eigen value) captures not only the connectivity of a node but also that of nodes adjacent to it, and nodes adjacent to its neighbours, and so on [34,35]. Thus, if *x*_*i*_ is the weight of the vertex *i*, then

$$ {x}_i=\frac{1}{\lambda }{\displaystyle \sum_{j=1}^n}{A}_{ij}{x}_j $$

where λ is a constant. This can be written in the matrix form as

$$ AX=\lambda X $$

where, *X* is the eigenvector of adjacency matrix, *A*, with eigenvalue λ. Thus *x*_*i*_, which depends on both the number and quality of connections, is proportional to the average of the centrality of its adjacent neighbours and is called the *eigenvector centrality* of the graph. It assigns relative scores to all the nodes in the network based on the principle that connections to high scoring nodes contribute more. In our earlier work, we carried out a comparative analysis of various graph centrality measures to identify tandemly repeated structural motifs. We observed that the principal eigenvector of the adjacency matrix was able to reliably capture the repetitive pattern of the structural units [36]. Below we present our proposed approach based on this eigenvector centrality.

### Dataset

In this study, a total of 125 Ankyrin repeat proteins were manually collected from Pfam [37], PROSITE (release 20.103) [38] and UniProt (release 2014_05) databases [39], and 5 designed Ankyrin proteins from the SCOP database (release 1.75) [40]. This is a redundant set with one or more structures corresponding to the same UniProt sequence. From this set, the training dataset of 58 proteins was constructed by considering the structure with highest resolution and maximum sequence coverage from each cluster of proteins corresponding to unique UniProt entries. For testing the performance of the algorithm, a total set of 370 proteins was taken comprising 125 known ANK repeat proteins (positive test set) and 245 non-solenoid proteins (negative test set), the benchmark dataset used by ConSole (http://console.sanfordburnham.org/GT/index.html). The complete set of 98,341 protein structures from Protein Data Bank (as of June 17, 2014) [41] was downloaded for detecting new Ankyrin repeat proteins.

### Algorithm

**Figure 3:**
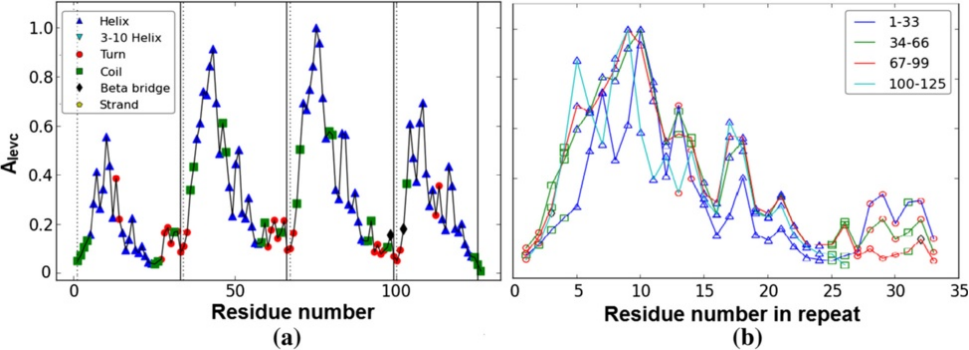
**Plot of principal eigenvectors of the adjacency matrix (** ***A***_***levc***_ **) for designed protein 1N0R. (a)** The principal eigenvectors of the adjacency matrix (*A*_*levc*_) for designed protein 1N0R is shown. The start and end of each repeat are indicated by dotted and solid lines respectively. **(b)** The overlap of *A*_*levc*_ profile for the repeat regions is shown. The points in different shapes correspond to the secondary structure elements.

The typical architecture of Ankyrin repeat motif is helix-turn-helix with the two helices anti-parallel and followed by a long variable loop which ends in a beta-turn, shown in Figure 1(a,b) and the eigenvector components of the principal eigenvalue of the adjacency matrix (*A*_*levc*_) is shown in Figure 1(c). The two peaks marked in Figure 1(c) fall within the helix regions and their large *A*_*levc*_ values are due to dense connections in the compact helical structures. A designed protein 1N0R (chain A) with four ANK repeats is shown in Figure 2(a) and its protein contact network in Figure 2(b). The 3-dimensional topology is well captured by the *A*_*levc*_ profile for each repeat unit as shown in Figure 3(a) for protein 1N0R. Based on secondary structure annotation as provided by STRIDE [42], we observe that both the helices and loop regions exhibit significant variation in their lengths as a result of insertions/deletions and is summarized in Table 1.

**Table 1:** **Characteristic features of Ankyrin repeat proteins**

**Length**	**1** ^**st**^ **helix**	**turn/coil**	**2** ^**nd**^ **helix**	**H-T-H core**	**Dist. between peak positions**	**ANK motif**
**Maximum**	12	8	21	34	15	49
**Minimum**	3	1	4	13	5	19
**Mean**	7.7	2.4	9.8	20	8.6	31.2

Variations in the length of the secondary structure elements and the distance between two peaks in the *A*_*levc*_ profile of the ANK motif for a dataset of 58 non-redundant protein structures is summarized.

The algorithm proceeds by parsing the STRIDE secondary structure assignment along the length of protein to look for helix-turn-helix motif (*H*_1_*TH*_2_) satisfying the following criteria:

1. The two helices are anti-parallel. Since the lengths of the two helices in ANK motif are comparable, a simple distance measure ${d}_{S_1{E}_2}<{d}_{E_1{E}_2}$ is used to confirm that the two helices are anti-parallel; *S*_1_: start of *H*_1;_*E*_1_, *E*_*2*_: end of *H*_1_, *H*_2_.
2. In the *A*_*levc*_ profile, ${A}_{H_1}>{A}_{H_2}$, where ${A}_{H_1}$, ${A}_{H_2}$: average magnitude of *A*_*levc*_ in *H*_1_, *H*_2_. This condition confirms that the first helix, *H*_1_, of the ANK motif is buried inside while the second helix, *H*_2_, lies on the outer surface.
3. Distance between the peak positions of the two helices in the *A*_*levc*_ profile ranges between 5 – 15 residues, to accommodate insertions/deletions in the individual secondary structure elements (Table 1).
4. Length of helix-turn-helix core is at least 13, as each helix is at least 4–6 residues and a coil in between of at least 2 residues. (Table 1).

Once the presence of an ANK motif is predicted by the above criteria, we define its start-end boundaries as follows. In earlier studies on constructing the consensus for designed ANK repeat proteins, the first helix is considered to start at the 5^th^ residue of the Ankyrin repeat [26]. We observed this to be true in 78% of ANK motifs annotated in the UniProt database (of the remaining 22%, ~53% are terminal repeats, which are generally incomplete). Hence, we define “- 4” position from the start of the 1^st^ helix (based on STRIDE annotation) as the *start* of the Ankyrin repeat. To define the end of the ANK motif, we look for a beta-turn from the end of the 2^nd^ helix to the start of the next repeat or 15 residues (whichever is lower). The position in the turn having the lowest *A*_*levc*_ value is considered as the end of the repeat region. In some cases the terminal repeat is at the end of the protein chain and has no β turn, in such cases the end of the protein is taken as the end of the terminal repeat. Finally, if at least two contiguous ANK repeats are identified within a threshold distance (≤17, half of a typical ANK motif), tandem ANK repeat region is reported.

### Implementation details

We have developed a web server for the identification of tandem repeats in protein structures by implementing the above algorithm. Python scripts are developed to automate the entire process involving construction of protein contact network, computing the eigen spectra [43], and obtaining secondary structure assignment using STRIDE. An implementation of the algorithm with a simple graphical output is deployed as a Web server for detecting ANK repeats, named as Ankyrin Repeat Identification by Graph Spectral Analysis (AnkPred) and is freely accessible at: http://bioinf.iiit.ac.in/AnkPred/. A user can enter a PDB Id or upload a structure in PDB format as input and identify ANK repeats by choosing the chain (default A). The output of the tool gives the number of predicted repeat copies, the coordinates of the repeat units and the 3-dimensional structure of the protein with each repeat unit highlighted in a different color, the non-repeat region being in grey. The overall complexity of the algorithm is O(2|*n*| + |*e*| + |*h*|), where *n* is the number of nodes (*C*_*α*_ atoms) in the network, *e* the number of edges in the network and *h* the total number of helices in the protein structure. The algorithm is computationally very efficient and the time taken to analyze the dataset of 370 proteins on a Intel(R) Core™ i5 processor with 4GB RAM is ~ 6 and ½ minutes.

## Results and discussion

**Figure 4:**
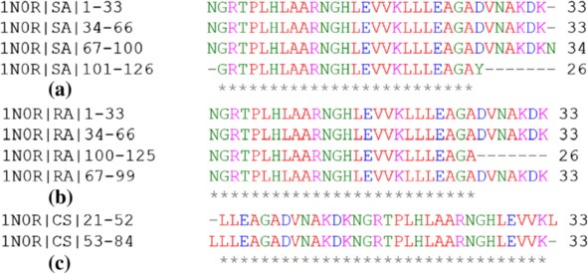
**MSA of the predicted repeat regions for 1N0R. (a)** predicted by the proposed approach, **(b)** RADAR output, and **(c)** ConSole output.

Here we present the analysis of the proposed algorithm on a representative set of fifteen ANK repeat proteins (Table 2). We first discuss in detail our analysis on a designed ANK protein, 1N0R (chain A), comprising four exact ANK repeats in tandem as shown in Figure 2(a) and its protein contact network given in Figure 2(b). The principal eigenvectors of the adjacency matrix, *A*_*levc*_, for designed ANK protein 1N0R is plotted in Figure 3(a). A clear repetitive pattern in the *A*_*levc*_ profile is observed in the four repeat regions (dashed and solid vertical lines correspond to start-end repeat boundaries based on RADAR output). This is clearly seen by overlapping the *A*_*levc*_ profile for the individual repeat copies in Figure 3(b) after normalizing with the largest peak in each repeat copy. The prediction is good both in terms of the copy number and start-end boundaries of the repeat regions compared with the sequence-based tool RADAR (see Table 2), while two repeat copies are missed by the structure based program ConSole, even in the case of designed ANK protein. The multiple sequence alignments (MSA) of the repeat regions predicted by our approach, RADAR and ConSole are shown in Figure 4(a), (b) and (c) respectively using CLUSTALW [44]. The MSA of the individual copies in both the cases is very well-conserved and in good agreement.

**Table 2:** **Prediction of repeat regions for a representative set of 15 proteins compared with UniProt annotation, RADAR and ConSole output**

**Protein name**	**PDB (chain)**	**UniProt annotation**	**RADAR**	**ConSole**	**AnkPred**
3ANK	1N0Q (A)	-	3-35, 36–68, 69-92	21-52	3-33, 34–67, 68-93
4ANK	1N0R (A)	-	1-33, 34–66, 67–99, 100-125	21-52, 53-84	1-33, 34–66, 67–100, 101-126
Protein phosphatase 1 regulatory subunit 12A	1S70 (B)	39-68, 72–101, 105–134, 138–164, 198–227, 231-260	47-73, 77–106, 110–139, 203–232, 236-265	44-74, 75–105, 106–136, 137–167, 175–205, 206–236, 237-267	36-71, 72–104, 105–138, 198–231, 232-267
Mouse GABP α/βdomain	1AWC (B)	5-34, 37–66, 70–99, 103–132, 136-166	13-34, 40–67, 73–100, 106-133	16-47, 48–79, 80-111	5-36, 37–69, 70–103, 104–136, 137-157
TRPV6 Ankyrin repeat domain	2RFA (A)	44-74, 78–107, 116–145, 162–191, 195–236, 238-267	46-91, 93–129, 164-208	117-132	44-77, 78–114, 116–142, 162–194, 195–237, 238-265
Yeast Nas6p complex with proteasome subunit, rpt3	2DZN (A)	1-30, 35–64, 71–100, 106–135, 139–168, 173-203	5-37, 38–70, 74–106, 109–141, 142-175	13-44, 45–76, 77–108, 112–143, 144–175, 176-207	3-34, 35–70, 71–105, 106–138, 139–172, 173–207, 208-228
D34 of human Ankyrin-R	1 N11 (A)	403-432, 436–465, 469–498, 502–531, 535–564, 568–597, 601–630, 634–663, 667–696, 700–729, 733–762, 766-795	406-431, 438–458, 471–487, 504–530, 537–563, 570–596, 603–629, 636–662, 669–695, 702–728, 735-761	415-446, 447–478, 479–510, 511–542, 543–574, 575–606, 607–638, 639–670, 671–702, 703–734, 735–766, 767-802	405-432, 436–458, 469–487, 503–535, 536–567, 568–600, 601–633, 634–666, 667–699, 700–733, 734–766, 767-796
Human gankyrin	1UOH (A)	3-36, 37–69, 70–102, 103–135, 136–168, 169–201, 202-226	49-78, 82–111, 115–144, 148–177, 181-210	23-53, 54–84, 85–115, 116–146, 147–177, 178-208	5-38, 39–71, 72–105, 106–137, 138–170, 171–204, 205-226
P53-53BP2 complex	1YCS (B)	958-990, 991-1023	959-987, 992-1020	337-352, 398-413	926-957, 958–990, 991–1024, 1025-1067
Tumor Suppressor P15(INK4B)	1D9S (A)	5-34, 38–66, 71–100, 104-130	24-44, 56–86, 88-119	78-111	71-104, 105-129
Notch	2F8Y (A)	1927-1956, 1960–1990, 1994–2023, 2027–2056, 2060-2089	1884-1930, 1931–1963, 1964–1997, 1998–2030, 2031–2063, 2064-2096	1919-1950, 1951–1982, 1983–2014, 2015–2046, 2047–2078, 2079-2110	1928-1960, 1961–1994, 1995–2027, 2028–2060, 2061–2094, 2095-2122
S. Cerevisiae Swi6 Ankyrin repeat fragmen	1SW6 (A)	318-346, 347–383, 384–469, 470–498, 499-514	348-391, 467-507	316-323, 324–331, 470-477	-
Human Osteoclast Stimulating Factor	3EHQ (A)	72-101, 105–135, 139-168	94-126, 128-159	83-114, 115–147, 148-180	72-104, 105–138, 139-177
Ankyrin repeat domain of Huntingtin interacting protein 14	3EU9 (A)	89-118, 123–155, 156–188, 189–219, 224-253	64-90, 94–123, 128–157, 161–190, 194–225, 229-252	70-101, 102–133, 134–165, 166–197, 198–229, 230-261	57-88, 89–122, 123–156, 157–189, 190–223, 224–257, 258-281
ANKRA	3SO8 (A)	148-180, 181–213, 214–246, 247–279, 280-313	34-89, 100-155	29-60, 61–92, 93–124, 125-156	149-180, 181–213, 214–246, 247–280, 281-310

**Figure 5:**
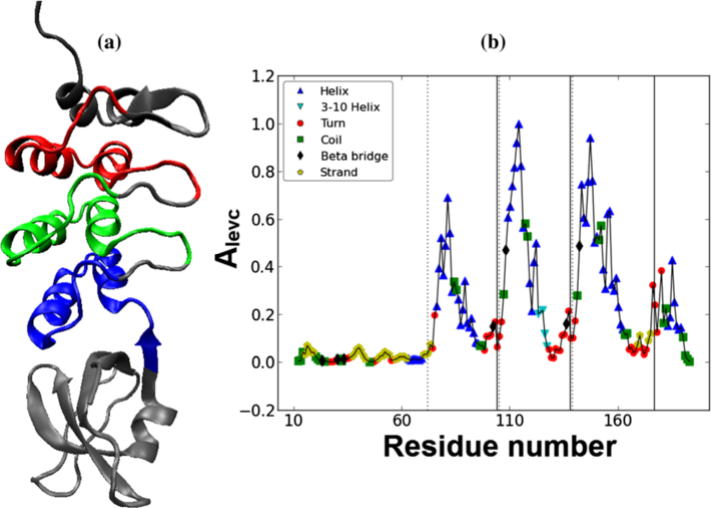
**Natural Ankyrin repeat protein 3EHQ (chain A). (a)** The 3d structure, and **(b)** the eigenvector components corresponding to the largest eigenvalue of adjacency matrix (*A*_*levc*_).

**Figure 6:**
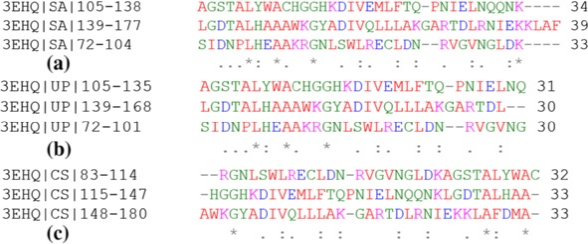
**MSA of the repeat regions in protein 3EHQ. (a)** predicted by the proposed approach, **(b)** annotated in the UniProt database, and **(c)** predicted by ConSole output.

We next consider an example of a natural protein, Osteoclast-stimulating factor 1, 3EHQ (chain A), that induces bone resorption. According to the annotation in UniProt, it contains three Ankyrin repeats from 72–168 as shown in the 3-D structure by different colours in Figure 5(a). In Figure 5(b) is shown the *A*_*levc*_ profile plot for 3EHQ, clearly indicating the presence of three repeating units in the region 72–177. There is a good agreement between the predicted start-end boundaries of the three repeat units with the UniProt annotation (see Table 2). However, the prediction of the repeat regions by RADAR and ConSole are not in accordance with the UniProt annotation. The RADAR prediction differs both in terms of the copy number and the repeat boundaries, the first repeat being completely missed. ConSole predicts three copies of the ANK repeats, but the positions of the start-end boundaries of the repeating units are off by about 10 residues for each repeat copy. In Figure 6 is shown the MSA of the repeat regions (a) predicted by our approach, (b) annotated in the UniProt database, and (c) predicted by ConSole. The MSA of the predicted repeat region in Figure 6(a) is in very good agreement with that of the UniProt annotated repeat regions (Figure 6(b)), compared to that of the ConSole predicted region in Figure 6(c). The results for a representative set of 15 ANK repeat proteins is summarized in Table 2 along with the annotation provided in UniProt database, and predictions by sequence and structure based methods, RADAR and ConSole, respectively. By and large we observe a good agreement in the detection of Ankyrin repeats both in copy number as well as repeat boundaries with UniProt annotation and also with ConSole.

**Figure 7:**
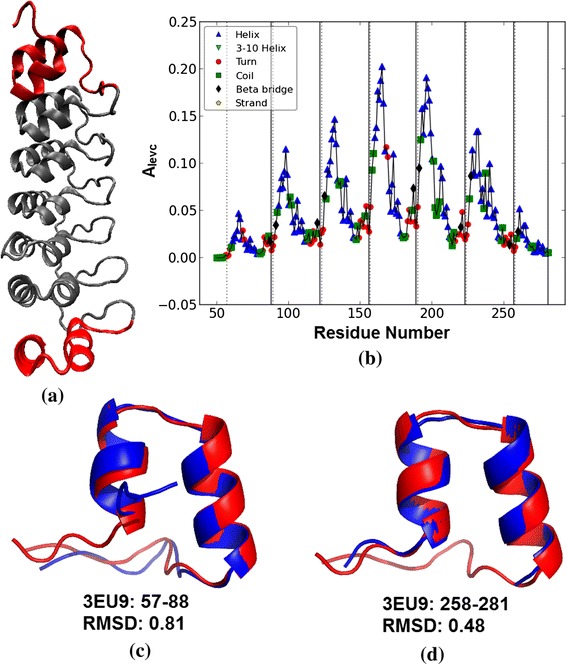
**Natural Ankyrin repeat protein 3EU9 (chain A). (a)** 3-D structure **(b)** Plot of the principal eigenvector of the adjacency matrix. **(c)** - **(d)** Structural alignment of extra Ankyrin repeat copy predicted in 3EU9 (shown in blue colour) with a repeat copy of designed protein 1N0R (shown in red colour).

**Figure 8:**
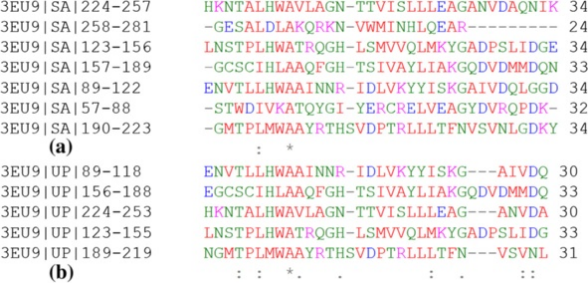
**MSA of the repeat regions in protein 3EU9. (a)** predicted by the proposed approach, and **(b)** annotated in UniProt database.

In Table 2 the proteins have been selected to present examples both of good agreement and of disagreement. Below we discuss a few examples in which our prediction differs from the annotation in UniProt database. For example, in the case of protein 3EU9 (chain A), five copies of ANK motifs are annotated in UniProt from 89–253, while our approach predicts seven copies, an extra copy on either side from 57–88 and 258–281. From the 3-D structure of 3EU9 in Figure 7(a) and *A*_*levc*_ profile shown in Figure 7(b), it is clear that the predicted terminal repeats (shown in red) exhibit *A*_*levc*_ profile similar to the five intermediate repeats (shown in gray). The structural alignment of these predicted terminal repeats with a representative structural ANK motif (from designed protein 1N0R) using Cealign module in Pymol [45] is shown in Figure 7(c) and (d); the Root Mean Square Deviation (RMSD) for each terminal copy is less than 1 Å indicating high structural similarity with the ANK motif. However at the sequence level these terminal repeats are not well conserved as is clear from the MSA of the predicted regions in Figure 8(a), compared to that of the UniProt annotated repeat regions in Figure 8(b). With one additional terminal copy predicted by ConSole, a total of six copies are predicted by it, but the boundaries of ConSole copies are shifted by around 10 residues as compared to UniProt annotation. In general, the terminal repeats are less conserved at the sequence level or incomplete, and their detection isn't easy. In 52 other proteins (see Additional file 1), additional copies of the ANK repeats have been predicted by the proposed approach, thus improving the annotation of the complete repeat region in these 53 proteins. In 16 of these cases, one extra copy is also predicted by ConSole. For the protein, 3SO8 (chain A, UniProt Id: Q9H9E1), initially three ANK repeats were annotated in the earlier release of UniProt (release 2012_08) from 181–279 whereas five repeats are predicted by our approach from residue 149–310, i.e., one extra repeat at each end. In the recent release of UniProt database (release 2014_05), the protein is now annotated as having five copies of the ANK motif from 148–313, which is in agreement with the prediction of the proposed approach (Table 2).

**Figure 9:**
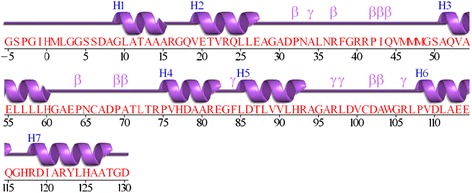
**Secondary structure representation of Ankyrin repeat protein 1D9S (chain A) from PDBsum.**

In protein 1D9S (chain A), four ANK repeats are reported from 5–130 in the UniProt database but only two are identified by our approach from 71–129. On analyzing the secondary structure architecture from PDBsum [46] for 1D9S in Figure 9, we observe that the region 38–66 contains only one helix assigned by both STRIDE [42] and DSSP [47] , while an ANK motif comprises of two anti-parallel helices, suggesting that this region may have been wrongly annotated in the UniProt database. The region 5–34 is predicted as ANK motif in the preliminary screening of our approach but is discarded in the post-processing step while reporting contiguous tandem repeat regions. A similar situation was encountered in 18 other proteins (see Additional file 1) where the first repeat in UniProt annotation is initially predicted by our algorithm, but later discarded because the next repeat is not identified within a threshold of 17 residues (half-length of an ANK motif). For all these proteins, except 4HBD, one or more copies are missed by ConSole as compared to UniProt annotation (see Additional file 1). It is possible that in all these proteins the missing ANK motif is mutated beyond recognition even at the structure level or there is a deletion of helix. Thus, we see that the eigen spectra of the adjacency matrix captures the repetitive fold pattern of the ANK motif very well and by incorporating the secondary structure information and variation in their lengths, an accurate prediction of the repeat boundaries is possible (Table 2). However, if there is an error in the secondary structure assignment, the prediction of the proposed algorithm is affected.

### Performance of the proposed algorithm

First, we discuss the prediction accuracy of ANK motifs with the UniProt annotation on a known set of 370 proteins comprising a positive test set of 125 Ankyrin repeat proteins and negative test set of 245 non-solenoid proteins. The results are summarized in Table 3 (a), where the sensitivity and specificity of the algorithm is computed as follows:

$$ Sensitivity=\frac{TP}{TP+FN}\simeq 0.976 $$

$$ Specificity=\frac{TN}{TN+FP}\simeq 1 $$

**Table 3:** **Performance of the proposed approach**

**a) Number of ANK proteins predicted on a dataset of 370 proteins (annotated in UniProt is 125)**					
**True positive**	**False positive**	**False negative**	**True negative**	**Sensitivity**	**Specificity**
122	0	3	245	0.976	1
**b) Number of ANK motifs predicted in 125 known ANK repeat proteins (annotated in UniProt is 584)**					
	**True positive**	**False positive**	**False negative**	**Sensitivity**	**Precision**
**AnkPred**	515	67	69	0.88	0.88
**ConSole**	419	109	165	0.72	0.79
**RADAR**	395	63	189	0.68	0.86

where *TP* corresponds to number of correctly predicted known Ankyrin repeat proteins, *FN* – the number of known Ankyrin repeat proteins missed by our approach, *FP* – the number of proteins predicted by our approach as containing tandem ANK repeats but not annotated as Ankyrin protein, and *TN* – the number of proteins correctly predicted by our approach as non-Ankyrin proteins. As there were only three false negatives (*FN*), 1SW6, 2ETB and 3ZRH, and no false positives (*FP*), the sensitivity and specificity of the algorithm is very high (≃1).

Next, for the predicted Ankyrin proteins, we analyze the number of ANK motifs correctly predicted in the dataset of 125 known Ankyrin repeat proteins and compare with a recent structure-based approach, ConSole, and a sequence based approach RADAR. In the UniProt database, a total of 584 ANK motifs are annotated in these 125 proteins, while 582 ANK motifs are predicted by the proposed approach, 528 by ConSole and 458 by RADAR. The details of the analysis are summarized in Table 3(b) in terms of sensitivity and precision, defined as:

$$ Sensitivity=\frac{TP}{TP+FN} $$

$$ Precision=\frac{TP}{TP+FP} $$

where, *TP* is the number of ANK motifs correctly predicted by the method in known dataset of 125 proteins, *FP* is the number of ANK motifs predicted by the method but not annotated in the UniProt database, and *FN* is the number of annotated ANK motifs missed by the method. It may be observed that both the sensitivity and precision of the proposed approach, AnkPred, is ~ 0.88, reasonably good compared to that of ConSole (0.72 and 0.79) and RADAR (0.68 and 0.86) respectively. The terminal copies are known to have low sequence conservation, resulting in lower sensitivity of the RADAR method. We recognise that the sensitivity of our algorithm, with its dependence on the secondary structure assignment, might be further improved.

To analyze the accuracy of the repeat boundaries predicted by the proposed approach, we constructed the *Multiple sequence alignment* (MSA) of the 582 predicted ANK motifs in the dataset of 125 known Ankyrin proteins using CLUSTALW [44].The consensus of the predicted ANK motifs was then built using SeaView [48] at 50% identity and is given below:

$$ \mathrm{XGXTPLHXAXXXGXXXXXXXLLXXXAXX} $$

This is in very good agreement with the consensus ANK motif proposed by Kohl *et al.* [49] and Mosavi *et al.* [50]. The conserved tetrapeptide motif TPLH at positions 4–7, Glycine at positions 2 and 13, and Leucine at positions 21–22 confirms the prediction accuracy of the repeat boundaries by the proposed approach.

### Analysis on protein data bank

**Figure 10:**
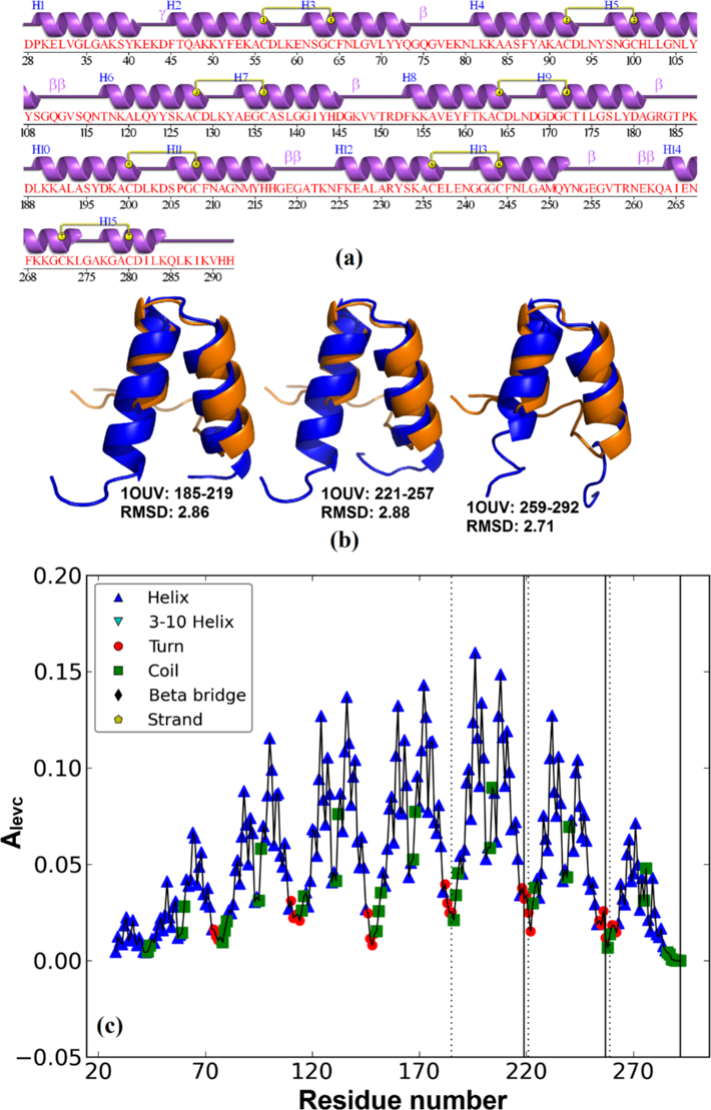
**Predicted Ankyrin repeat protein 1OUV (chain A). (a)** Secondary structure representation from PDBsum **(b)** Structural alignment of predicted ANK repeat copy (shown in blue colour) with a repeat copy of designed ANK protein 1N0R (shown in orange) **(c)** *A*_*levc*_ plot with dotted and solid lines showing the start and end of predicted ANK boundaries.

We performed the proposed algorithm on the complete PDB. A total number of 98,341 structures represented as proteins or proteins in complex with nucleic acids were downloaded. On removing short fragments < 50 residues (as these are unlikely to contain two contiguous copies of ANK motifs) and proteins with no secondary structures assigned, a total of 94,975 structures were used for analysis. The proposed algorithm identified 819 protein structures containing at least two tandemly repeated ANK motifs. Of these 181 are annotated as known ANK proteins in UniProt, Pfam, PROSITE and PDB of which ~ 50 structures contain designed Ankyrin repeat proteins (DARPINS). The number of correctly predicted Ankyrin repeat proteins is 178 and only 3 were missed by our approach, 1SW6 (chain A), 2ETB (chain A) and 3ZRH (chain A). In the first two cases the proposed approach missed the detection of ANK motifs as the UniProt annotated repeat regions contain 3–4 helices while according to rules defined in the algorithm, an ANK motif comprises of two anti-parallel helices. In 3ZRH the two annotated copies of ANK repeats are not contiguous but separated by 23 residues, and hence missed by our approach. Thus, the remaining 641 structures are proposed as previously unrecognised Ankyrin repeats and are listed in Additional file 2. It is observed that 27 of these proteins are annotated as containing other repeat types, *viz.*, 9 TPR, 7 Pumilio repeat, 2 HEAT, 2 Annexin repeat, 2 Tumor necrosis factor receptor (TNFR-Cys), 2 Mitochondrial termination factor repeat (MTERF), 2 Clathrin heavy chain repeat (CHCR) and 1 HAT (Additional file 2). Structurally, TPR, HEAT and HAT motifs are very similar to ANK repeat motif, each of them comprising two anti-parallel helices forming a Helix–Turn–Helix core and are also of similar lengths, ~ 30–34 residues. The major difference being the ANK motif has a long loop ending in a β turn which is not present in TPR, HEAT and HAT motifs. Even with such strong similarity between these structural motifs, only 13 false positives (9 TPR, 3 HEAT and 1 HAT) are reported by our approach. To check the reliability of our prediction in these proteins, we carried out structure-structure superposition of the predicted ANK repeat region with a DARPin motif from 1N0R using Cealign module in Pymol [45]. For example, in protein 1OUV (chain A), seven copies of TPR are reported in UniProt database from 29–278 (Additional file 2) containing 14 helices *H*_1_-*H*_14_ as shown in the secondary structure representation from PDBsum [46] in Figure 10(a). The superposition is good with root mean square deviation (RMSD) for all the three predicted ANK repeats units < 3 Å as shown in Figure 10(b). The *A*_*levc*_ profile in the Ankyrin predicted region from 185 to 292 in Figure 10(c) is also very similar to that for a typical ANK motif in Figure 1(a). In this case, the predicted ANK repeat motifs are within the TPR annotated region, comprised of one helix from each adjacent TPR repeats and can be represented as ${H}_2^i{T}^i{H}_1^{i+1}$ where ${H}_2^i$ is the second helix of the *i*^*th*^ TPR motif and ${H}_1^{i+1}$ is the first helix of the (*i* + 1)^*th*^ TPR motif. The structural alignment of the 7 annotated TPR regions was performed with a representative TPR motif from designed protein 1NA0 and RMSD for each repeat unit < 2 Å (results not shown) suggesting that UniProt annotation is also correct. However, the β turn between two helices within a TPR motif was observed to be longer than that of the typical designed TPR motif and resembling the terminal loop of the ANK motif. This suggests the possibility of multi-repeat architecture in complex proteins. For 21 other repeat proteins, a similar multi-repeat architecture was observed. In the case of HEAT repeat protein 3LWW (chain A), the annotation in UniProt is six continuous copies from 124–441 and two distant copies from 602–641 and 687–726. The predicted ANK repeat lies in the non-HEAT region from 520–621 with very small overlap of 20 residues with HEAT repeat. In this case two different repeats are present in different regions in the protein and a total of 10 proteins containing two different repeat types non-overlapping each other was observed (marked ‘*’ in Additional file 2). For these proteins that exhibit multi-repeat architecture, it would be interesting to analyze the interaction sites which would help in confirming multiple annotations/functions in these proteins with complex architecture. Thus, the structure based approach proposed here is promising in detecting tandem structural repeats in proteins and is powerful enough to distinguish between very similar structural repeats, *viz*. Ankyrin and TPR/HEAT/HAT.

### Functional analysis of previously unrecognized ankyrin proteins

We identified 641 previously unrecognized Ankyrin repeat proteins by the proposed approach. In Table 4, we present our analysis of 11 of these proteins. In all these proteins, we observe that the binding sites reported in PDBsum [46] lie in the predicted Ankyrin repeat region. For example, the DNA polymerase lambda protein 3HWT (Human), which is important for the DNA replication process, contains four domains. The reported DNA binding sites in 3HWT are present in DNA polymerase domain (257–331) and lie on the second helix of both the copies of predicted Ankyrin units. The presence of Ankyrin repeats in the DNA binding proteins, 1SW6 and 3V30, annotated in UniProt provides support to our prediction and possible functional role of 3HWT. This analysis helps in understanding the type of interaction 3HWT is involved in and comparison with other proteins with similar functions can lead to a better understanding of the role of Ankyrin repeats. Similarly, the interaction of Ankyrin repeats with RNA is known in the case of 1WDY and 4G8K. We observe that proteins 3Q0P, 3K4E and 3V71 have binding sites reported in the predicted repeat region with RNA as the binding partner, again providing support to our prediction.

**Table 4:** **Example proteins with binding sites in the predicted Ankyrin repeat region**

**PDB**	**UniProt**	**Organism**	**Predicted Ankyrin region**	**Binding partner in predicted region obtained from PDBsum**
3HWT	Q9UGP5	Homo sapiens	257-291, 292-319	DNA
1FO3	Q9UKM7	Homo sapiens	285-319, 324–381, 397–444, 458-504	Kifunensine and Sulphate
1KRF	P31723	Penicillium citrinum	263-308, 325-381	Kifunensine, N-Acetyl-D-Glucosamine and Mannose
2IQC	Q9NPI8	Homo sapiens	253-299, 300-341	Hg (Mercury)
3Q0P*	Q14671	Homo sapiens	894-923, 929–959, 965-995	RNA
3K4E*	Q07807	S. cerevisiae	539-576, 584-613	RNA
3V71*	O44169	C. elegans	203-230, 240-270	RNA
4F42*	P07174	R. norvegicus	19-46, 60-85	MNB
2VTB	Q84KJ5	A. thaliana	378-408, 412-445	FAD and MHF
3FY4	O48652	A. thaliana	343-378, 379-417	FAD, IMD and MES
4LCT	P45432	A. thaliana	221-252, 262-294	Sulphate

*Multi repeat protein where predicted repeat region is overlapping with other repeat annotated in UniProt.

We predicted Ankyrin repeats in two mannosidase protein structures, 1FO3 (human) and 1KRF (P. citrinum). Kifunensine (KIF) is the inhibitor of mannosidases and regulates the activity of these proteins. In PDBsum, the KIF binding sites for the proteins 1FO3 and 1KRF are annotated in the region predicted as Ankyrin repeat by our approach. This suggests novel interactions of these Ankyrin repeat proteins. Thus one could carry out a systematic analysis of other previously unrecognized Anyrin proteins to identify their interacting partners, leading to an understanding of their functional role.

### Analysis of modelled ankyrin proteins

**Figure 11:**
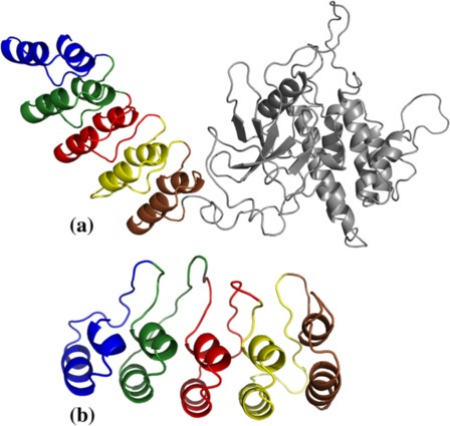
**Prediction on modelled structures shown. (a)** Integrin-linked protein kinase (UniProt Id: Q99J82). The repeat boundaries of five Ankyrin motifs predicted by AnkPred (shown in different colours) are in good agreement with five annotated copies in Uniprot. **(b)** ANKRD protein (UniProt Id: Q7Z3H0). In this case only 3 Ankyrin motifs are annotated in UniProt (intermediate copies) while AnkPred predicts two additional copies on either side.

Protein structural information is increasing at a rapid pace with advances in resolving protein structures, but is still not comparable to the wealth of sequence information. It may be noted that of over 1200 proteins annotated as containing Ankyrin repeat motifs in the UniProt database, only about 60 Ankyrin proteins have structural information available. To show the efficacy of our approach on modelled structures, we modelled 30 Ankyrin repeat proteins from UniProt database for which the structure is not yet resolved. The structures were modelled using Swiss-Model server [51], which identifies template structures from PDB [41] based on sequence coverage and sequence identity. The templates having high coverage and sequence identity in the repeat region are selected for homology-based modelling of these 30 protein sequences. The proposed algorithm, AnkPred, is executed on the corresponding modelled proteins and the prediction of repeat regions is given in the Additional file 3. In Figure 11(a) is shown the prediction of the proposed approach on the modelled structure of the Integrin-linked protein kinase (UniProt Id: Q99J82), which is in very good agreement with annotation in UniProt. It may be noted that in about half of the proteins (marked by an asterisk in Additional file 3), the copy number predicted had increased, with terminal repeats being identified. It is known that terminal copies are generally less conserved and sometime incomplete [26], and hence missed by sequence-based methods, but are identified by our structure-based method as shown for ANKRD (UniProt Id: Q7Z3H0) protein in Figure 11(b). This suggests the power of our approach in improving the annotation of repeat regions for protein sequences for which no structure information is available.

### Analysis of other structural repeats

**Figure 12:**
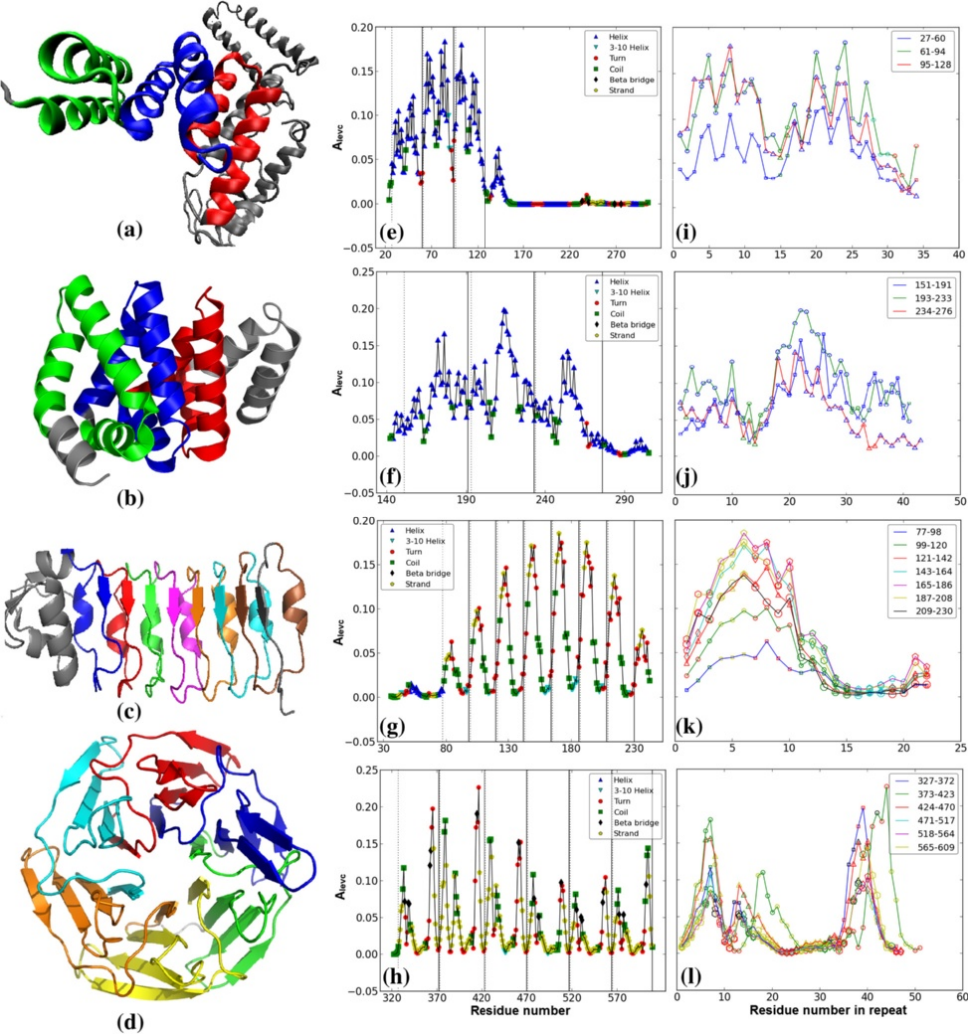
**Proteins of other structural repeat families. (a)**-**(d)** 3-D structure: **(a)** 2C2L: chain A (TPR) **(b)** 3SL9: chain A (ARM) **(c)** 1D0B: chain A (LRR) **(d)** 1U6D: chain X (KELCH). In **(e)**, **(f)**, **(g)** and **(h)** the *A*_*levc*_ plot for respective proteins shown. In **(i)**, **(j)**, **(k)** and **(l)** the *A*_*levc*_ profile of the repeat regions in respective proteins are overlapped.

To assess the efficacy of the proposed approach on other protein repeat families, we next present our analysis on four different repeat types: Tetratricopeptide repeat (TPR), Armadillo repeat (ARM), Leucine-rich repeat (LRR) and Kelch repeat. The 3-dimensional structure of a representative protein from each repeat type is shown in Figure 12(a)-(d) and their respective *A*_*levc*_ profiles in Figure 12(e)-(h). A unique *A*_*levc*_ profile is observed in the repeat regions in each of these proteins which are well conserved within the adjacent repeating units as depicted by overlapping the *A*_*levc*_ profile in the repeating units in Figure 12(i)-(l). The distinct *A*_*levc*_ profiles for different repeats correspond to the specific orientation of the secondary structural elements in each repeat type. It may be noted that the *A*_*levc*_ profile for the TPR repeat is very distinct compared to that of Ankyrin repeat (Figure 3(a)), although it is of similar length and has very similar secondary structure architecture with helix-turn-helix core. This clearly shows the power of the eigen spectra analysis of the protein contact network in the identification of structural repeats and its sensitivity in distinguishing similar structural repeats.

## Conclusions

Most proteins with tandemly repeated structural motifs of 20–50 residues length interact with other proteins. The identification of these repeats can be informative in understanding the structure and function of these proteins. Here we show that the structural repeat motifs exhibit a specific pattern in the eigen spectra profile of the adjacency matrix of a protein structure represented as a graph. Thus graph-spectral analysis provides an efficient tool in the detection of repeats of different shapes and forms. Analysis of the results suggest that the proposed method discovers more Ankyrin repeat proteins and repeats per protein than existing sequence and structure based methods. The annotation of the complete repeat region has been improved in 53 proteins and 641 previously unrecognized Ankyrin repeat proteins have been identified by the proposed approach. However, the performance of the proposed approach is affected by the assignment of secondary structures by STRIDE/DSSP; only if a secondary structure element is completely missed; the prediction accuracy is not affected by small insertions/deletions within secondary structure elements.

In our preliminary analysis of some of the newly predicted Ankyrin proteins we observe that the reported binding sites lie in the predicted repeat regions providing support to our prediction. A systematic analysis of these proteins can lead to the understanding of their interacting partners and help towards the functional annotation of these proteins. We also show that the proposed approach can be successfully used on modelled proteins for identification of repeats and can help in improving the annotation. Since a large number of proteins do not have any structural information, and sequence-based repeat detection methods are limited by detectable similarity in the repeating units, this analysis greatly enhances the capability of the algorithm. As shown it is possible to easily extend and automate the proposed approach for the identification of other protein repeat families by considering information available on known repeat families.

## Additional files

Additional file 1
**Comparison of the proposed approach with UniProt, RADAR and ConSole is shown for the set of 130 known Ankyrin repeat proteins with the best resolution structure for each UniProt entry highlighted in bold.**
Additional file 2
**Previously unrecognized Ankyrin proteins predicted by the proposed approach in Protein Data Bank.**
Additional file 3
**Prediction of Ankyrin repeats by the proposed approach is compared with the UniProt annotation for a set of 30 modelled protein structures.**
