# Supplementary material for: Identifying tandem Ankyrin repeats in protein structures
Source: BMC Bioinformatics. 2014 Dec 30;15(1):6599. doi: 10.1186/s12859-014-0440-9 (PMC4307672; doi:10.1186/s12859-014-0440-9)
Supplement: Additional file 1 — Comparison of the proposed approach with UniProt, RADAR and ConSole is shown for the set of 130 known Ankyrin repeat proteins with the best resolution structure for each UniProt entry highlighted in bold. [file 12859_2014_440_MOESM1_ESM.docx]

**Comparison of the proposed approach with UniProt, RADAR and ConSole is shown for the set of 130 known Ankyrin repeat proteins with the best resolution structure for each UniProt entry highlighted in bold.**

| **S. No** | **UniProt** | **PDB (chain)** | **UniProt annotation** | **RADAR** | **ConSole** | **AnkPred** |
| --- | --- | --- | --- | --- | --- | --- |
|  | Designed protein with 3 ANK | **1N0Q(A)** | - | 3-35, 36-68, 69-92 | 21-52 | 3-33, 34-67, 68-93 |
|  |  | 2ZGG(A) |  |  |  |  |
|  | Designed protein with 4 ANK | **1N0R(A)** | - | 1-33, 34-66,  67-99, 100-125 | 21-52, 53-84 | 1-33, 34-66,  67-100, 101-126 |
|  | Designed protein with 5 ANK | **1MJ0(A)** | - | 21-44, 47-77, 80-110, 113-143, 146-165 | 30-61, 62-93, 94-125 | 11-45, 46-78, 79-111, 112-145, 146-166 |
|  |  | 2QYJ(A) |  |  |  |  |
|  | **Q9Z2X2** (Gankyrin-S6ATPase) | **3AJI(A)** | 3-36, 37-69, 70-102, 103-135, 136-168, 169-201, 202-226 | 49-78, 82-111,  115-144, 148-177, 181-210 | 21-51, 52-82, 83-114, 115-145, 146-176, 177-207 | 5-38, 39-71, 72-104, 105-137, 138-170, 171-204, 205-231 |
|  |  | 2DWZ(C) |  |  |  |  |
|  |  | 2DVW(A) |  |  |  |  |
|  | **Q9Y5S1** (Human TRPV2) | **2F37(B)** | 72-114, 115-161, 162-207, 208-243, 244-292, 293-319 | 147-217, 231-302 | 80-125, 126-170, 171-215, 216-261, 262-307 | 162-200, 208-244, 245-292, 293-318 |
|  | **Q9WUD2** (Ankyrin repeat domain of TRPV2) | **2ETA(A)** | 73-115, 116-162, 163-208, 209-244, 245-293, 294-320 | 160-186, 200-232 | 119-126, 161-168, 169-176, 210-217, 218-225, 230-237, 299-306 | 163-200, 209-244, 245-293, 294-319 |
|  |  | 2ETB^†^(A) |  |  |  |  |
|  |  | 2ETC^†^(A) |  |  |  |  |
|  | **Q9HBA0** (Human TRPV4 ankyrin repeat domain) | **4DX1*****(A)** | 237-266, 284-313, 369-398 | 90-153, 173-238 | 162-207, 208-253, 254-299, 303-348 | 237-275, 284-319, 320-369, 370-397 |
|  |  | 4DX2*(A) |  |  |  |  |
|  | **Q9H9E1** (ANKRA) | **3V31(A)** | 148-180, 181-213, 214-246, 247-279, 280-313 | 175-230, 241-296 | 157-188, 189-220, 221-252, 253-284, | 149-180, 181-213, 214-246, 247-280, 281-310 |
|  |  | 3V2O(A) |  |  |  |  |
|  |  | 3V2X(A) |  |  |  |  |
|  |  | 3SO8(A) |  |  |  |  |
|  |  | 4LG6(A) |  |  |  |  |
|  | **Q9H9B1** (EuHMT1 (Glp) Ankyrin Repeat Domain) | **3B7B(A)** | 737-766, 772-801, 805-834, 838-868, 872-901, 905-934, 938-967 | 754-779, 787-812, 820-846, 854-879, 887-912, 920-945 | 747-778, 779-810, 811-842, 843-874, 875-906, 907-938 | 741-774, 775-806, 807-841, 842-874, 875-906, 907-940, 941-967 |
|  |  | 3B95(A) |  |  |  |  |
|  | **Q9H2K2** (Human Tankyrase 2 in complex with human 3BP2) | **3TWR*****(A)** | 525-557, 558-590, 591-623 | 1. 48-59, 78-92  2. 61-75, 94-108, 127-141  3.110-126, 143-158 | 532-562, 564-594, 595-625 | 490-523, 525-557, 558-590, 591-624, 625-646 |
|  |  | 3TWQ*(A) |  |  |  |  |
|  |  | 3TWS*(A) |  |  |  |  |
|  |  | 3TWT*(A) |  |  |  |  |
|  |  | 3TWU*(A) |  |  |  |  |
|  |  | 3TWV*(A) |  |  |  |  |
|  |  | 3TWW*(A) |  |  |  |  |
|  |  | 3TWX*(A) |  |  |  |  |
|  | **Q9DFS3** (Chicken TRPV4 Ankyrin repeat domain) | **3JXI*(A)** | - | 223-286, 306-371 | 142-187, 188-233, 234-279, 289-334 | 223-261, 270-305, 306-354, 355-384 |
|  |  | 3JXJ(A) |  |  |  |  |
|  | **Q99728** (BARD1 Ankyrin Repeat Domain) | **3C5R(A)** | 427-459, 460-492, 493-525, 526-546 | 425-452, 455-485, 488-518, 521-545 | 440-470, 471-501, 502-532 | 427-459, 460-486, 493-526, 527-546 |
|  | **Q978J0** (Thermophilic Ankyrin Repeat Protein) | **2RFM*****(A)** | 31-60, 64-93, 97-126, 130-159 | 26-47, 51-80, 84-113, 117-146, 150-178 | 17-48, 49-80, 81-112, 113-144, 145-176 | 31-63, 64-96, 97-129, 130-162, 163-189 |
|  | **Q96NW4** (Ankyrin repeat domain of Varp complex with Vamp7 domain) | **4B93***(B) | 743-772, 776-805, 809-838, 842-871 | 1. 86-118, 119-151, 152-184  2. 187-215, 220-246 | 818-847, 848-877 | 743-775, 776-808, 809-841, 842-875, 876-895 |
|  | **Q96DX5** (Human ASB9) | **3D9H*****(A)** | 35-64, 68-97,  101-130, 133-162, 166-195, 198-227 | 55-85, 88-118,  121-151, 153-183, 186-216, 218-247 | 45-75, 76-106,  107-137, 138-168, 169-199, 200-230 | 35-67, 68-101,  102-132, 133-165, 166-192, 198-231, 232-253 |
|  |  | 2XAI*(D) |  |  |  |  |
|  |  | 3ZKJ(D) |  |  |  |  |
|  |  | 3ZNG***(A)** |  |  |  |  |
|  | **Q92882** (Human Osteoclast Stimulating Factor) | **3EHQ(A)** | 72-101, 105-135, 139-168 | 94-126, 128-159 | 83-114, 115-147, 148-180 | 72-104, 105-138, 139-177 |
|  |  | 3EHR(A) |  |  |  |  |
|  | **Q91WD2** (mouse TRPV6 Ankyrin repeat domain) | **2RFA(A)** | 44-74, 78-107,  116-145, 162-191, 195-236, 238-267 | 46-91, 93-129,  164-208 | 117-132 | 44-77, 78-114,  116-142, 162-194, 195-237, 238-265 |
|  | **Q90623** (Protein phosphatase 1 regulatory subunit 12A) | **1S70(B)** | 39-68, 72-101,  105-134, 138-164, 198-227, 231-260 | 47-73, 77-106,  110-139, 203-232, 236-265 | 44-74, 75-105,  106-136, 137-167, 175-205, 206-236, 237-267 | 36-71, 72-104,  105-138, 198-231, 232-267 |
|  | **Q8WUF5** (C-terminal region of human IASPP) | **2VGE*****(A)** | 659-691, 692-724 | 1. 638-667, 670-700  2. 724-740, 766-782 | 633-664, 665-696, 697-728 | 623-658, 659-691, 692-726, 727-758 |
|  | **Q8TDY4** (Arf6 in complex with ASAP3) | **2B0O*****(E)** | 585-617, 621-650 | 622-650, 655-681 | 553-587, 588-622, 623-657 | 621-654, 655-684 |
|  |  | 3LVQ*(E) |  |  |  |  |
|  |  | 3LVR*(E) |  |  |  |  |
|  | **Q8K424** (Transient receptor potential cation channel subfamily V member 3) | **4N5Q(A)** | 214-243, 261-291, 340-369, | 262-285, 341-364 | 177-222, 223-268, 269-314 | 214-251, 261-294, |
|  | **Q8IUH5** (Ankyrin repeat domain of Huntingtin interacting protein 14) | **3EU9*****(A)** | 89-118, 123-155, 156-188, 189-219, 224-253 | 64-90, 94-123,  128-157, 161-190, 194-225, 229-252 | 70-101, 102-133,  134-165, 166-197, 198-229, 230-261 | 57-88, 89-122,  123-156, 157-189, 190-223, 224-257, 258-281 |
|  | **Q838Q8** (EF0377) | **3HRA(A)** | - | 4-29, 30-63, 64-96, 97-130, 131-169, 170-197 | 20-51, 52-83, 84-115, 116-147 | 4-37, 38-70, 71-104, 105-139, 140-177, 178-201 |
|  | **Q6PFX9** (Mouse Tankyrase-Axin complex) | **3UTM*****(B)** | 361-390, 394-423, 427-456, 514-546, 550-579, 583-612 | 51-83, 84-116,  117-149, 240-272, 273-305 | 338-372, 373-407, 408-442, 443-477, 478-512, 518-552, 553-587, 588-622 | 328-360, 361-393, 394-427, 428-460, 461-507, 550-583, 584-616 |
|  | **Q6IV60** (Host-range protein K1 in Vaccinia virus) | **3KEA**(A) | - | 13-47, 76-111 | 21-52, 53-84, 86-118, 119-152, 153-187, 188-220, 223-255 | 2-28, 29-59, 60-92, 93-126, 193-220, 221-251 |
|  | **Q63ZY3** (KANK2 ankyrin repeats) | **4HBD**^†^(A) | 666-696, 700-733, 738-767, 771-801, 805-835 | 74-127, 130-164, 167-199, 202-232, 235-266 | 641-675, 676-710, 711-745, 746-780, 781-815 | 738-770, 771-805, 806-832 |
|  | **Q60778** (IKBBETA/NF-KB P65 Homodimer Complex) | **1OY3(D)** | 57-86, 93-122,  126-155, 206-235, 240-269, 273-302 | 57-85, 90-119,  203-232, 237-266, 270-298 | 64-75 | 57-92, 93-125,  126-148, 206-239, 240-273, 274-304 |
|  |  | **1K3Z(D)** |  |  |  |  |
|  | **Q60773** (p19INK4d/ Cdk6 complex) | **1BLX***(B) | 41-69, 73-102,  106-135, 138-165 | 36-96, 101-161 | 17-47, 48-78, 79-109, 110-140 | 6-41, 42-72, 73-105, 106-138, 139-164 |
|  |  | 1AP7^†^(A) |  |  |  |  |
|  | **Q5ZXN6** (Legionella pneumophila FIC domain complex with cytidine monophosphate) | **4BES*****(A)** | 391-420, 424-453 | 1. 19-50, 65-108, 117-144  2. 328-357, 360-386 | 424-432, 433-441 | 348-384, 391-423, 424-464 |
|  |  | 4BER*(A) |  |  |  |  |
|  |  | 4BEP*(A) |  |  |  |  |
|  |  | 4BET*(A) |  |  |  |  |
|  | **Q15027** (ArfGAP and ANK repeat domain of ACAP1) | **3JUE(A)** | 606-635, 639-668, 672-702 | 613-644, 646-677 | 433-464, 577-608, 609-640, 641-672 | 606-639, 640-672, 673-697 |
|  |  | 3T9K*(B) |  |  |  |  |
|  |  | 4F1P(A) |  |  |  |  |
|  | **Q13625** (P53-53BP2 complex) | **1YCS***(B) | 958-990, 991-1023 | 959-987,992-1020 | 337-352, 398-413 | 926-957, 958-990, 991-1024, 1025-1067 |
|  |  | 4A63*(B) |  |  |  |  |
|  | **Q13418** (Ankyrin repeat domain of integrin-linked kinase) | **4HI8(A)** | 2-30, 31-63, 64-96, 97-129, 130-174 | 45-73, 78-107,  111-140 | 12-43, 44-75, 76-107, 108-139 | 2-24, 33-65, 66-93, 99-132, 133-167 |
|  |  | 2KBX(A) |  |  |  |  |
|  |  | 3IXE(A) |  |  |  |  |
|  |  | 3F6Q(A) |  |  |  |  |
|  |  | 4HI9(A) |  |  |  |  |
|  | **Q05823** (Sensor domain of human RNase L) | **4G8K(A)** | 24-53, 58-87, 91-120, 124-153, 167-197, 201-234, 238-268, 272-301, 303-329 | 29-73, 74-139,  140-217, 221-287, 288-336 | 31-38, 63-70, 96-103, 126-133, 134-141, 174-181, 239-246, 247-254, 270-277, 278-285, 309-316 | 21-57, 58-90, 91-123, 124-158, 167-200, 201-233, 238-272, 273-300, 301-327 |
|  |  | 1WDY(A) |  |  |  |  |
|  |  | 4G8L(B) |  |  |  |  |
|  |  | 4OAU(C) |  |  |  |  |
|  |  | 4OAV(B) |  |  |  |  |
|  | **Q01705** (Mouse Notch 1 Ankyrin Repeat Intracellular Domain) | **2QC9*****(A)** | 1917-1946,  1950-1980,  1984-2013,  2017-2046,  2050-2079 | 1918-1950,  1951-1982,  1985-2017,  2018-2050,  2051-2083 | 1924-1955,  1990-2020,  2021-2051,  2053-2083 | 1917-1949,  1950-1983,  1984-2016,  2017-2049,  2050-2083,  2084-2106 |
|  |  | 1YMP(A) |  |  |  |  |
|  | **Q00420** (Mouse GABP α/β domain) | **1AWC(B)** | 5-34, 37-66, 70-99, 103-132, 136-166 | 13-34, 40-67, 73-100, 106-133 | 16-47, 48-79, 80-111 | 5-36, 37-69, 70-103, 104-136, 137-157 |
|  | **P62775** (Myotrophin) | **1MYO(A)** | 2-30, 34-66, 67-99 | 8-32, 38-65, 71-100 | 24-55, 56-87 | 1-35, 36-66, 67-100, |
|  |  | 2MYO(A) |  |  |  |  |
|  | **P62774** (V-1 bound to capping protein) | **2KXP(C)** | 2-30, 34-66, 67-99 | 8-32, 38-65, 71-100 | 616-647 | 1-35, 36-66, 67-94 |
|  | **P58546** (Actin capping protein in complex with V-1) | **3AAA*****(C)** | 2-30, 34-66, 67-99 | 8-32, 38-65, 71-100 | 42-73, 74-105 | 2-33, 34-67, 68-100, 101-118 |
|  | **P55273** (Cyclin-dependent kinase 4 inhibitor D) | **1BD8*****(A)** | 41-69, 73-102,  106-135, 138-166 | No repeats found | 17-46, 47-76, 77-106, 107-136 | 7-41, 42-72, 73-105, 106-138, 139-162 |
|  |  | 1BI8*(B) |  |  |  |  |
|  | **P55271** (Tumor Suppressor P15 - INK4B) | **1D9S**^†^(A) | 5-34, 38-66, 71-100, 104-130 | 24-44, 56-86, 88-119 | 78-111 | 71-104, 105-129 |
|  | **P50086** (Yeast Nas6p complex with the proteasome subunit, rpt3) | **2DZN***(A) | 1-30, 35-64, 71-100, 106-135, 139-168, 173-203 | 5-37, 38-70, 74-106, 109-141, 142-175 | 13-44, 45-76, 77-108, 112-143, 144-175, 176-207 | 3-34, 35-70, 71-105, 106-138, 139-172, 173-207, 208-228 |
|  |  | 1WG0*(A) |  |  |  |  |
|  |  | 1IXV*(A) |  |  |  |  |
|  |  | 2DZO(A) |  |  |  |  |
|  | **P46683** (Ankyrin repeat-containing protein YAR1) | **4BSZ***(B) | 49-78, 92-121, | 52-84, 90-120,  124-154 | 21-28, 31-38, 73-80, 99-106 | 7-48, 49-91, 92-126, 127-156, |
|  | **P46531** (Human Notch1 ankyrin repeat) | **2F8Y*****(A)** | 1927-1956,  1960-1990,  1994-2023,  2027-2056,  2060-2089 | 1884-1930,  1931-1963,  1964-1997,  1998-2030,  2031-2063,  2064-2096 | 1919-1950,  1951-1982,  1983-2014,  2015-2046,  2047-2078,  2079-2110 | 1928-1960,  1961-1994,  1995-2027,  2028-2060,  2061-2094,  2095-2122 |
|  |  | 2F8X*(K) |  |  |  |  |
|  |  | 1YYH*(A) |  |  |  |  |
|  |  | 2HE0*(A) |  |  |  |  |
|  |  | 3NBN*(B) |  |  |  |  |
|  |  | 3V79*(K) |  |  |  |  |
|  | **P42773** (F71N mutant of p18INK4c) | **1IHB***^†^(A) | 4-33, 37-65, 69-98, 102-132 | 13-43, 48-76, 77-108, 111-140 | 48-82, 83-117 | 69-101, 102-136, 137-160 |
|  |  | 1G3N*(B) |  |  |  |  |
|  |  | 1BU9*(A) |  |  |  |  |
|  |  | 1MX2*^†^(A) |  |  |  |  |
|  |  | 1MX4*^†^(A) |  |  |  |  |
|  |  | 1MX6(A) |  |  |  |  |
|  | **P42771** (CDK6-P16INK4A Tumor Suppressor complex) | **1BI7**^†^(B) | 11-40, 44-72, 77-106, 110-139 | 25-58, 59-87, 91-125 | 29-60, 76-107 | 77-110, 111-134 |
|  |  | 1A5E^†^(A) |  |  |  |  |
|  |  | 1DC2^†^(A) |  |  |  |  |
|  |  | 2A5E^†^(A) |  |  |  |  |
|  | **P25963** (Ikappa-B-alpha/NF-kappa-B complex) | **1IKN*(D)** | 73-103, 110-139, 143-172, 182-211, 216-245 | 73-101, 102-133, 134-166, 167-205, 206-239 | 83-118, 119-149, 150-180, 185-215, 218-248, 249-279 | 110-142, 143-172, 182-215, 216-249, 250-286 |
|  |  | 1NFI*(E) |  |  |  |  |
|  | **P20749** (Ankyrin repeat domain of Bcl-3: IkappaB protein family) | **1K1A(A)** | 134-163, 171-200, 204-235, 241-270, 275-304, 308-337, 338-367 | 156-188, 189-221, 226-258, 259-292, 293-325 | 135-166, 167-198, 199-230, 231-262, 263-294, 295-326 | 126-162, 163-195, 196-233, 234-267, 268-300, 301-333, 334-352 |
|  |  | 1K1B(A) |  |  |  |  |
|  | **P16157** (D34 of human Ankyrin-R) | **1N11**(A) | 403-432, 436-465, 469-498, 502-531, 535-564, 568-597, 601-630, 634-663, 667-696, 700-729, 733-762, 766-795 | 406-431, 438-458, 471-487, 504-530, 537-563, 570-596, 603-629, 636-662, 669-695, 702-728, 735-761 | 415-446, 447-478, 479-510, 511-542, 543-574, 575-606, 607-638, 639-670, 671-702, 703-734, 735-766, 767-802 | 405-432, 436-468, 469-501, 502-535, 536-567, 568-600, 601-633, 634-666, 667-699, 700-733, 734-766, 767-796 |
|  | **P14585** (CSL-Notch-Mastermind ternary complex bound to DNA) | **2FO1*****(E)** | 1093-1122,  1126-1158,  1162-1194,  1206-1236,  1240-1269 | 1. 1214-1232, 1248-1266  2. 1083-1113, 1115-1147  3. 1149-1166, 1181-1198  4. 1200-1211, 1234-1245 | 1039-1069,  1070-1102,  1103-1134,  1135-1170,  1219-1251,  1252-1283 | 1052-1086,  1094-1125,  1161-1201,  1207-1239,  1242-1273,  1274-1297 |
|  | **P09959** (S. Cerevisiae Swi6 Ankyrin repeat fragment) | **1SW6**^†^**(A)** | 318-346, 347-383, 384-469, 470-498, 499-514 | 348-391, 467-507 | 316-323, 324-331, 470-477 | - |
|  | **P07207** (Ankyrin Domain of the Drosophila Notch Receptor) | **1OT8(A)** | 1950-1979,  1983-2013,  2017-2046,  2050-2079,  2083-2112,  2116-2139 | 1936-1964,  1970-1996,  2004-2031,  2037-2064,  2070-2093 | 39-70,  71-102,  103-134,  135-166,  167-198 | 1950-1982,  1983-2016,  2017-2035,  2050-2082,  2083-2116,  2117-2137 |
|  | **O75832** (Human gankyrin) | **1UOH(A)** | 3-36, 37-69, 70-102, 103-135, 136-168, 169-201, 202-226 | 49-78, 82-111,  115-144, 148-177, 181-210 | 23-53, 54-84, 85-115, 116-146, 147-177, 178-208 | 5-38, 39-71, 72-105, 106-137, 138-170, 171-204, 205-226 |
|  |  | 1TR4(A) |  |  |  |  |
|  |  | 1QYM(A) |  |  |  |  |
|  | **O35433** (Ankyrin repeat domain of TRPV1) | **2NYJ**^†^(A) | 110-152, 153-199, 200-246, 247-282, 283-331, 332-358 | 213-249, 278-333 | 253-260, 340-347 | 200-237, 247-282, 283-332, 333-359 |
|  |  | 2PNN^†^(A) |  |  |  |  |
|  |  | 3J5P^†^(A) |  |  |  |  |
|  |  | 3J5Q^†^(B) |  |  |  |  |
|  |  | 3J5R^†^(A) |  |  |  |  |
|  | **O22265** (chloroplast signal recognition particle protein cpSRP43) | **3DEO(A)** | 136-158, 159-188, 193-222 | 168-198, 202-231 | 138-168 | 159-192, 193-226 |
|  |  | 3DEP(A) |  |  |  |  |
|  |  | 3UI2(A) |  |  |  |  |
|  | **O14593** (Ankyrin Repeat Domains of Human RFXANK) | **3V30(A)** | 89-118, 123-152,  156-185, 189-218, 222-251 | 29-61, 62-94, 95-127, 128-150 | 118-149, 150-181, 182-213, | 92-122, 123-155,  156-189, 190-222, 223-252 |
|  |  | 3UXG(A) |  |  |  |  |
|  | **E9ADW8** (Ankyrin repeat protein from Leishmania major) | **3LJN(A)** | - | 1. 24-41, 88-105  2. 137-177, 210-248 | 28-62, 63-98,  137-172, 173-211 | 207-239, 240-276 |
|  | **P51480** (Cyclin-dependent kinase inhibitor 2A) | **1LNN*****(A)** | 3-32, 36-64, 69-98, 102-131 | 1. 11-38, 43-71  2. 85-111, 118-144 | 30-61, 62-93, 94-125 | 1-32, 33-63, 64-97, 98-131, 132-155 |
|  | **Q9UGI0** (Ubiquitin thioesterase ZRANB1) | **3ZRH**^†^**(A)** | 260-290, 313-340 | 1. 466-490, 494-516  2. 395-404, 643-652 | 319-326, 575-582 | - |

*Extra copy of repeat identified by proposed approach

^†^Initially predicted UniProt copy is discarded in post-processing step
