# Supplementary material for: Identifying tandem Ankyrin repeats in protein structures
Source: BMC Bioinformatics. 2014 Dec 30;15(1):6599. doi: 10.1186/s12859-014-0440-9 (PMC4307672; doi:10.1186/s12859-014-0440-9)
Supplement: Additional file 2 — Previously unrecognized Ankyrin proteins predicted by the proposed approach in Protein Data Bank. [file 12859_2014_440_MOESM2_ESM.docx]

## Previously unrecognized Ankyrin proteins predicted by the proposed approach in Protein Data Bank.

| **PDB** | **Chain** | **UniProt Id** | **UniProt Annotation** | **UniProt repeat type** | **Predicted Ankyrin region** | **Predicted copy number** |
| --- | --- | --- | --- | --- | --- | --- |
| 1OUV | A | O25728 | 29-62, 64-98, 100-133, 134-170, 172-205,  206-242, 244-278 | TPR | 185-219, 221-257, 259-292 | 3 |
| 4N3A | A | O15294 | 21-54, 89-122, 123-156, 157-190, 191-224,  225-258, 259-292,  293-326, 327-360,  361-394, 395-428,  429-462, 463-473 | TPR | 381-417, 431-471 | 2 |
| 1KLX | A | O25103 | 1-28, 57-94, 97-130 | TPR | 73-107, 109-135 | 2 |
| 2Q7F | B | O34452 | 1-23, 24-57, 59-91, 93-125, 127-159, 160-193 | TPR | 156-192, 206-236 | 2 |
| 4KVO | B | O74985 | 10-43, 45-77, 78-111,  146-182, 185-218,  380-413, 415-447,  493-526, 677-710 | TPR | 142-180, 181-216, 217-254 | 3 |
| 2GW1 | A | P07213 | 99-132, 134-165, 281-315, 363-396, 397-430,  432-464, 465-498,  505-541, 542-575 | TPR | 342-377, 378-411 | 2 |
| 4JSN | B | P42345 | 1346-1382, 1383-1408, 1409-1442, 1443-1473, 1474-1507, 1508-1541, 1542-1574, 1575-1614, 1615-1649, 1650-1693, 1694-1731, 1732-1786, 1787-1846, 1898-1930, 1931-1970, 1971-2005 | TPR | 1470-1502, 1503-1539 | 2 |
| 2J9Q | B | P50542 | 335-368, 369-402,  403-436, 452-485,  488-521, 522-555,  556-589 | TPR | 552-594, 595-639 | 2 |
| 3HYM | J | Q13042 | 130-163, 299-333,  334-367, 368-401,  403-435, 445-478,  479-512 | TPR | 367-400, 401-439 | 2 |
| 2Q4C | A | Q9SYT0 | 15-80, 87-152, 170-236, 246-311 | Annexin | 9-68, 69-100 | 2 |
| 1W7B | A | P07355 | 42-102, 114-174, 199-259, 274-334 | Annexin | 89-121, 122-153 | 2 |
| 3LVG | B | P49951 | 537-683, 686-828,  833-972, 979-1124,  1128-1269, 1274-1420, 1423-1566 | CHCR | 1354-1388, 1391-1414,  1415-1443, 1446-1486 | 4 |
| 3LVH | A |  |  |  | 1310-1353, 1354-1388,  1391-1418 | 3 |
| 2OND | A | Q99LI7 | 45-77, 79-110, 117-152, 163-196, 221-261,  271-303, 319-352,  354-387, 458-494 | HAT | 336-369, 370-405 | 2 |
| 2Z5N | A | Q92973 | 132-169, 178-215,  219-256, 401-438,  442-479, 484-521,  568-607, 671-708 | HEAT | 490-531, 532-577 | 2 |
| 3K7V | A | P30153 | 8-46, 47-84, 85-123,  124-161, 162-200,  201-239, 240-278,  279-321, 322-360,  361-399, 400-438,  439-477, 478-516,  517-555, 556-589 | HEAT | 452-490, 491-530 | 2 |
| 4FP9 | B | Q7Z6M4 | 142-172, 177-204,  209-239, 245-270,  290-318 | MTERF | 216-240, 249-284, 294-327 | 3 |
| 4FZV | B |  |  |  | 249-280, 294-328 | 2 |
| 3Q0P | A | Q14671 | 848-883, 884-919,  920-955, 956-991,  992-1027, 1028-1063, 1064-1099, 1103-1142 | Pumilio | 894-923, 929-959, 965-995 | 3 |
| 3BSX | B |  |  |  | 885-923, 929-959 | 2 |
| 1M8X | B |  |  |  | 893-923, 929-959 | 2 |
| 3Q0M | B |  |  |  | 885-923, 928-959 | 2 |
| 3Q0N | A |  |  |  | 848-887, 893-923 | 2 |
| 3K4E | C | Q07807 | 538-573, 574-609,  610-645, 646-681,  682-717, 718-759,  760-795, 807-844 | Pumilio | 539-576, 584-613 | 2 |
| 3V71 | A | O44169 | 86-124, 128-163, 164-200, 201-236, 237-279,  287-324, 326-361,  370-411 | Pumilio | 203-230, 240-270 | 2 |
| 4F42 | A | P07174 | 32-65, 67-108, 109-147, 149-189 | TNFR-Cys | 19-46, 60-85 | 2 |
| 4F44 | B |  |  |  | 19-46, 60-85 | 2 |
| 1NGR* | A |  |  |  | 364-392, 393-418 | 2 |
| 3LWW* | A | Q14974 | 124-163, 168-207,  213-250, 317-357,  362-399, 404-441,  602-641, 687-726 | HEAT | 520-570, 573-621 | 2 |
| 3O2Q* | D | Q92797 | 31-64, 67-101, 104-146, 153-192, 227-266 | HEAT | 246-286, 287-340 | 2 |
| 4H3H* | A |  |  |  | 246-287, 288-340 | 2 |
| 4H3K* | D |  |  |  | 246-286, 287-340 | 2 |
| 3GB8* | A | O14980 | 217-240, 241-277,  354-472, 515-553,  560-597, 602-639,  775-813, 885-916,  917-954, 1002-1039 | HEAT | 678-734, 739-794 | 2 |
| 4EBA* | B | Q6CII8 | 62-94, 99-133, 143-179, 190-223, 264-296,  305-337, | HAT | 447-481, 482-514 | 2 |
| 4HOQ* | A | Q13325 | 51-84, 94-127, 138-173, 181-214, 249-282,  338-371, 376-410, 435-468 | TPR | 261-305, 306-352 | 2 |
| 4BUJ* | F | P17883 | 4-37, 47-80, 425-458,  471-507, 508-541,  627-661, 702-735,  736-769, 945-985,  987-1018, 1226-1259 | TPR | 1367-1389, 1391-1420 | 2 |
| 4HNY* | C | P12945 | 20-53, 54-87, 91-124,  126-162, 241-274,  384-417, 452-485, 728-761 | TPR | 783-820, 823-862 | 2 |
| 4CR3 | T | P32496 |  |  | 3-45, 51-92, 97-131 | 3 |
| 4CR4 | T |  |  |  | 3-47, 50-96, 97-131 | 3 |
| 4CR2 | T |  |  |  | 3-45, 51-95 | 2 |
| 2NZ7 | B | Q9Y239 |  |  | 23-48, 60-97 | 2 |
| 4E9M | E |  |  |  | 23-48, 60-99 | 2 |
| 4IQJ | C | Q9XDH5 |  |  | 832-874, 887-926, 928-974 | 3 |
| 2HPI | A |  |  |  | 832-874, 887-926 | 2 |
| 3E0D | A |  |  |  | 832-874, 887-926 | 2 |
| 1EJJ | A | Q9X519 |  |  | 364-392, 396-450 | 2 |
| 1EQJ | A |  |  |  | 364-392, 396-450 | 2 |
| 1O98 | A |  |  |  | 364-392, 396-450 | 2 |
| 1O99 | A |  |  |  | 364-393, 396-450 | 2 |
| 1Z77 | A | Q9X0C0 |  |  | 78-143, 144-200 | 2 |
| 2ID6 | A |  |  |  | 102-143, 144-200 | 2 |
| 3IH2 | A |  |  |  | 78-143, 144-200 | 2 |
| 3IH3 | A |  |  |  | 43-105, 107-170 | 2 |
| 3IH4 | A |  |  |  | 78-143, 144-200 | 2 |
| 3O3F | A | Q9X017 |  |  | 119-152, 153-185 | 2 |
| 3O3G | A |  |  |  | 119-152, 153-185 | 2 |
| 3H37 | A | Q9WZH4 |  |  | 289-340, 341-381, 388-429 | 3 |
| 3H38 | A |  |  |  | 289-340, 341-386, 388-424 | 3 |
| 3H39 | A |  |  |  | 289-340, 341-381, 389-424 | 3 |
| 3H3A | A |  |  |  | 289-340, 341-378, 388-426 | 3 |
| 2HPG | D | Q9WYF8 |  |  | 246-304, 306-335 | 2 |
| 1FO3 | A | Q9UKM7 |  |  | 285-319, 324-381,  397-444, 458-504 | 4 |
| 1X9D | A |  |  |  | 324-381, 397-445 | 2 |
| 3G2S | B | Q9UJY5 |  |  | 38-99, 100-128 | 2 |
| 4HXI | A | Q9UH77 |  |  | 115-149, 150-177 | 2 |
| 3HWT | A | Q9UGP5 |  |  | 257-291, 292-319 | 2 |
| 1FNT | f | Q9U8G2 |  |  | 31-67, 68-101 | 2 |
| 1DGM | A | Q9TVW2 |  |  | 141-159, 164-199 | 2 |
| 3U57 | A | Q9SPP9 |  |  | 104-141, 149-186 | 2 |
| 3U5U | A |  |  |  | 104-141, 149-186 | 2 |
| 3U5Y | B |  |  |  | 104-141, 149-186 | 2 |
| 4ATD | A |  |  |  | 104-141, 149-186 | 2 |
| 4ATL | A |  |  |  | 104-141, 149-186 | 2 |
| 4EK7 | B |  |  |  | 104-141, 149-186 | 2 |
| 3ZJ6 | A |  |  |  | 104-141, 149-186 | 2 |
| 4A3Y | A |  |  |  | 104-141, 149-186 | 2 |
| 3OND | A | Q9SP37 |  |  | 392-433, 449-482 | 2 |
| 2VF8 | A | Q9RYW8 |  |  | 172-203, 204-233 | 2 |
| 2A4M | A | Q9RVD6 |  |  | 58-95, 98-119 | 2 |
| 1YIA | C |  |  |  | 58-95, 98-119 | 2 |
| 1YID | C |  |  |  | 58-95, 98-119 | 2 |
| 1VW2 | I | Q9RN43 |  |  | 441-473, 480-514 | 2 |
| 2FV0 | A | Q9RC92 |  |  | 50-86, 94-126 | 2 |
| 4I6G | A | Q9R194 |  |  | 352-388, 389-424 | 2 |
| 4MLP | A |  |  |  | 352-385, 388-423 | 2 |
| 3OX5 | A | Q9NZU7 |  |  | 40-77, 92-114 | 2 |
| 1JUQ | C | Q9NZ52 |  |  | 37-98, 99-127 | 2 |
| 1LF8 | A |  |  |  | 37-98, 99-127 | 2 |
| 2IQC | A | Q9NPI8 |  |  | 253-299, 300-341 | 2 |
| 3TEF | A | Q9KMU2 |  |  | 266-286, 302-329 | 2 |
| 4LZ6 | A | Q9KAX3 |  |  | 270-351, 365-413 | 2 |
| 3J1T | A | Q9JHU4 |  |  | 3350-3373, 3374-3427 | 2 |
| 4CEM | A | Q9HAU5 |  |  | 164-200, 214-255, 256-300 | 3 |
| 1UW4 | B |  |  |  | 858-909, 912-967 | 2 |
| 2Y9U | A | Q9H3D4 |  |  | 545-576, 577-611 | 2 |
| 4IFP | A | Q9C000 |  |  | 370-399, 411-459 | 2 |
| 3F07 | B | Q9BY41 |  |  | 18-60, 69-105 | 2 |
| 4IFG | A | Q9BJF5 |  |  | 225-268, 269-292 | 2 |
| 3N0G | A | Q9AR86 |  |  | 116-138, 144-177,  184-225, 239-264 | 4 |
| 3N0F | B |  |  |  | 144-177, 184-225 | 2 |
| 4B3K | A | Q99YP9 |  |  | 128-155, 162-213 | 2 |
| 4B3L | B |  |  |  | 128-155, 162-213 | 2 |
| 2KK0 | A | Q99856 |  |  | 75-100, 114-145 | 2 |
| 3MOP | C | Q99836 |  |  | 43-66, 67-104 | 2 |
| 3M7G | A | Q977W1 |  |  | 96-133, 136-168 | 2 |
| 3M6K | B |  |  |  | 96-133, 136-168 | 2 |
| 3M6Z | A |  |  |  | 96-133, 136-168 | 2 |
| 3M7D | A |  |  |  | 96-132, 136-168 | 2 |
| 2CSB | A |  |  |  | 96-132, 136-163 | 2 |
| 1WY6 | A | Q970G9 |  |  | 7-39, 40-76 | 2 |
| 1OU5 | A | Q96Q11 |  |  | 273-311, 325-354 | 2 |
| 2VTV | A | Q939Q9 |  |  | 207-230, 235-265 | 2 |
| 2X5X | A |  |  |  | 207-230, 235-265 | 2 |
| 2X76 | A |  |  |  | 207-230, 235-265 | 2 |
| 4BYM | A |  |  |  | 207-230, 235-265 | 2 |
| 4BRS | A |  |  |  | 207-230, 235-265 | 2 |
| 4BVJ | A |  |  |  | 207-230, 235-265 | 2 |
| 4BTV | A |  |  |  | 207-230, 235-265 | 2 |
| 4BVK | A |  |  |  | 207-230, 235-265 | 2 |
| 1FNN | A | Q8ZYK1 |  |  | 129-155, 164-184 | 2 |
| 2ZBL | B | Q8ZKT7 |  |  | 106-161, 167-213 | 2 |
| 3FPK | A | Q8ZKP5 |  |  | 142-177, 181-205 | 2 |
| 3G05 | A | Q8XAY0 |  |  | 445-490, 505-534 | 2 |
| 3SQV | A | Q8X5G6 |  |  | 360-396, 411-443 | 2 |
| 2KG5 | A | Q8WWN8 |  |  | 25-56, 57-86 | 2 |
| 2LYQ | A | Q8VL32 |  |  | 13-37, 39-65 | 2 |
| 2LYS | A |  |  |  | 13-36, 39-64 | 2 |
| 1B25 | A | Q8U1K3 |  |  | 379-414, 418-453 | 2 |
| 1B4N | D |  |  |  | 379-409, 418-453 | 2 |
| 2A8Z | A | Q8RJN8 |  |  | 16-56, 73-119 | 2 |
| 1Z2Z | A | Q8Q0M2 |  |  | 177-207, 208-242 | 2 |
| 1YW0 | A | Q8PDA8 |  |  | 45-121, 126-151 | 2 |
| 3E08 | A |  |  |  | 42-121, 125-151 | 2 |
| 3BK9 | D |  |  |  | 42-120, 125-151 | 2 |
| 2XGM | B | Q8PC69 |  |  | 87-123, 140-172 | 2 |
| 2DI3 | B | Q8NLM6 |  |  | 91-133, 134-183 | 2 |
| 1WMG | A | Q8K1S3 |  |  | 876-900, 915-943 | 2 |
| 3RC3 | A | Q8IYB8 |  |  | 564-614, 622-689 | 2 |
| 3J61 | o | Q8H5N0 |  |  | 139-180, 183-206 | 2 |
| 3N70 | H | Q8FAN8 |  |  | 223-245, 255-275 | 2 |
| 2ZC2 | B | Q8DT97 |  |  | 125-153, 154-198 | 2 |
| 4F66 | A | Q8DT00 |  |  | 139-166, 175-199 | 2 |
| 1X0P | B | Q8DMN3 |  |  | 1094-1117, 1118-1140 | 2 |
| 2FI0 | A | Q8CZ42 |  |  | 11-38, 48-81 | 2 |
| 2K9Q | B | Q8ABY1 |  |  | 14-38, 39-68 | 2 |
| 3D3A | A | Q8AB22 |  |  | 47-85, 90-118 | 2 |
| 2R5S | A | Q87RI8 |  |  | 212-245, 249-284 | 2 |
| 3IGY | B | Q86N96 |  |  | 386-415, 418-472 | 2 |
| 3IGZ | B |  |  |  | 386-414, 418-472 | 2 |
| 2VTB | E | Q84KJ5 |  |  | 378-408, 412-445 | 2 |
| 2EF8 | A | Q83VS9 |  |  | 26-51, 52-88 | 2 |
| 3CLQ | A | Q82ZN9 |  |  | 188-222, 228-264 | 2 |
| 3SQN | A | Q82ZN7 |  |  | 2-29, 30-68 | 2 |
| 1TZ9 | B | Q82ZC9 |  |  | 242-273, 287-323 | 2 |
| 1MIW | A | Q7SIB1 |  |  | 303-339, 340-360, 361-404 | 3 |
| 4HH7 | A | Q7BVV5 |  |  | 8-47, 62-101 | 2 |
| 1VU9 | K | Q79791 |  |  | 158-192, 193-228 | 2 |
| 1VUA | L |  |  |  | 158-193, 194-229 | 2 |
| 1VUO | T |  |  |  | 107-156, 157-207 | 2 |
| 1VUQ | 2 |  |  |  | 107-157, 158-208 | 2 |
| 1VUX | T |  |  |  | 107-157, 158-206 | 2 |
| 1VUZ | Q |  |  |  | 157-192, 193-216 | 2 |
| 1VV9 | 2 |  |  |  | 158-191, 192-220 | 2 |
| 3J34 | S |  |  |  | 107-157, 158-206 | 2 |
| 3VXC | A | Q76BU9 |  |  | 366-393, 401-435 | 2 |
| 2K5E | A | Q74DN8 |  |  | 7-30, 39-73 | 2 |
| 2J7A | O | Q72EF4 |  |  | 36-60, 62-100 | 2 |
| 2B43 | B | Q6REV3 |  |  | 431-474, 475-506 | 2 |
| 2JBR | A | Q6Q272 |  |  | 36-82, 89-139 | 2 |
| 2JBS | A |  |  |  | 36-82, 89-139 | 2 |
| 2JBT | A |  |  |  | 36-82, 89-139 | 2 |
| 2ENK | A | Q6PML9 |  |  | 13-45, 50-101 | 2 |
| 3R3U | C | Q6NAM1 |  |  | 250-269, 278-299 | 2 |
| 3N1X | A | Q6IV66 |  |  | 437-459, 460-492 | 2 |
| 3N1Z | A |  |  |  | 437-458, 460-491 | 2 |
| 3L2F | F | Q6AW42 |  |  | 150-179, 183-207 | 2 |
| 2LS7 | A | Q62048 |  |  | 13-53, 56-90 | 2 |
| 3GOZ | A | Q5ZSW6 |  |  | 30-58, 59-87, 88-116,  117-145, 146-174,  175-203, 204-234 | 7 |
| 3VUQ | C | Q5SLX6 |  |  | 103-147, 148-187 | 2 |
| 2Z07 | B | Q5SJN0 |  |  | 296-342, 352-399 | 2 |
| 3AUO | A | Q5SJ64 |  |  | 293-314, 315-347 | 2 |
| 4EV0 | A | Q5SID7 |  |  | 98-159, 162-203 | 2 |
| 2EPG | B | Q5SHE5 |  |  | 149-176, 177-202 | 2 |
| 2ZEJ | A | Q5S007 |  |  | 1417-1444, 1450-1489 | 2 |
| 3DPJ | B | Q5LS67 |  |  | 98-140, 141-187 | 2 |
| 3GZS | B | Q5L9X2 |  |  | 49-85, 102-142 | 2 |
| 3CE2 | A | Q5L5N2 |  |  | 260-314, 324-367 | 2 |
| 3PGY | A | Q5HE87 |  |  | 313-350, 357-389 | 2 |
| 1YGM | A | Q5BU39 |  |  | 8-64, 65-112 | 2 |
| 1EGM | M | Q59472 |  |  | 46-73, 79-127, 129-171 | 3 |
| 1EGV | M |  |  |  | 46-73, 79-127, 129-171 | 3 |
| 1UC4 | M |  |  |  | 46-73, 79-127, 129-171 | 3 |
| 1DIO | G |  |  |  | 79-127, 129-171 | 2 |
| 1EEX | G |  |  |  | 79-127, 129-171 | 2 |
| 1IWB | G |  |  |  | 79-127, 129-171 | 2 |
| 1UC5 | G |  |  |  | 79-127, 129-171 | 2 |
| 3AUJ | L | Q59470 |  |  | 441-466, 473-510 | 2 |
| 4MMI | A | Q59289 |  |  | 311-346, 350-382 | 2 |
| 3MM5 | A | Q59109 |  |  | 357-387, 395-417 | 2 |
| 3MM6 | A |  |  |  | 357-387, 395-417 | 2 |
| 3MM7 | A |  |  |  | 357-389, 395-417 | 2 |
| 3MM8 | A |  |  |  | 357-387, 395-417 | 2 |
| 3MM9 | A |  |  |  | 357-389, 395-417 | 2 |
| 3MMA | A |  |  |  | 357-389, 395-417 | 2 |
| 3MMB | D |  |  |  | 357-387, 395-417 | 2 |
| 3MMC | A |  |  |  | 357-387, 395-417 | 2 |
| 3I05 | A | Q580R7 |  |  | 110-147, 150-185 | 2 |
| 4HEA | C | Q56221 |  |  | 19-68, 84-114 | 2 |
| 2EHW | C | Q53WA3 |  |  | 2-33, 35-73 | 2 |
| 4H7A | A | Q53VY0 |  |  | 78-109, 124-156 | 2 |
| 2ZCA | A |  |  |  | 78-109, 124-156 | 2 |
| 3V93 | A | Q53I60 |  |  | 520-558, 562-611 | 2 |
| 3DKW | I | Q51441 |  |  | 109-171, 177-220 | 2 |
| 3ALR | D | Q4QRE8 |  |  | 90-114, 118-144 | 2 |
| 3P0H | A | Q4QFJ7 |  |  | 413-450, 453-490 | 2 |
| 3P0J | C |  |  |  | 413-450, 453-489 | 2 |
| 3ATQ | A | Q4JA33 |  |  | 369-401, 402-452 | 2 |
| 1E6V | B | Q49601 |  |  | 194-231, 246-290, 296-350 | 3 |
| 3CF4 | A | Q46G04 |  |  | 698-745, 749-779 | 2 |
| 1DK5 | A | Q42657 |  |  | 35-75, 76-107 | 2 |
| 1V02 | A | Q41290 |  |  | 107-143, 152-189 | 2 |
| 1V03 | A |  |  |  | 107-143, 152-189 | 2 |
| 2ONG | B | Q40322 |  |  | 102-149, 150-192 | 2 |
| 2ONH | A |  |  |  | 102-149, 150-183 | 2 |
| 2YPF | A | Q3ZD73 |  |  | 435-467, 469-504,  605-649, 650-674 | 4 |
| 4GJR | B | Q3ZD72 |  |  | 423-456, 457-490, 491-540 | 3 |
| 4GG4 | A |  |  |  | 389-422, 423-470 | 2 |
| 4GJP | A |  |  |  | 491-524, 525-573 | 2 |
| 3GLQ | A | Q3JY79 |  |  | 382-421, 437-470 | 2 |
| 2ZZR | A | Q3DUP2 |  |  | 119-163, 171-218 | 2 |
| 3G4D | A | Q39761 |  |  | 82-105, 107-140 | 2 |
| 3G4F | A |  |  |  | 82-105, 107-140 | 2 |
| 2P1N | D | Q39255 |  |  | 91-125, 126-160 | 2 |
| 3OGK | G |  |  |  | 91-125, 126-160 | 2 |
| 3OGM | G |  |  |  | 91-125, 126-160 | 2 |
| 3NVL | A | Q38AH1 |  |  | 385-413, 417-470 | 2 |
| 3S9V | B | Q38710 |  |  | 285-307, 311-349, 365-397 | 3 |
| 3N58 | A | Q2YQX8 |  |  | 375-414, 430-463 | 2 |
| 3QB2 | A | Q2VJ58 |  |  | 64-109, 111-156 | 2 |
| 4G1K | A | Q2SZN7 |  |  | 169-216, 231-251 | 2 |
| 3GPK | A | Q2G9Z0 |  |  | 240-260, 264-294 | 2 |
| 3MKR | A | Q28104 |  |  | 212-247, 263-307 | 2 |
| 2NOX | A | Q1LK00 |  |  | 60-138, 142-168 | 2 |
| 3IVP | A | Q180H4 |  |  | 50-77, 78-117 | 2 |
| 2QY7 | A | Q14677 |  |  | 20-55, 72-120 | 2 |
| 4A64 | A | Q13620 |  |  | 448-492, 493-533 | 2 |
| 2EE4 | A | Q13017 |  |  | 97-129, 130-179 | 2 |
| 2PE4 | A | Q12794 |  |  | 93-119, 133-161 | 2 |
| 3K8P | D | Q12745 |  |  | 337-383, 395-431 | 2 |
| 2VK4 | B | Q12629 |  |  | 216-252, 261-291 | 2 |
| 3KT8 | A | Q12109 |  |  | 140-178, 181-217 | 2 |
| 2RFQ | B | Q0S811 |  |  | 6-51, 58-107 | 2 |
| 3ZKW | B | Q0P8Q4 |  |  | 245-264, 280-310 | 2 |
| 1H2T | C | Q09161 |  |  | 458-489, 494-522 | 2 |
| 3VR3 | B | Q08636 |  |  | 480-543, 545-584 | 2 |
| 1BXL | A | Q07817 |  |  | 105-133, 135-185 | 2 |
| 3W5E | A | Q07343 |  |  | 391-435, 436-485 | 2 |
| 3MQE | B | Q05769 |  |  | 460-483, 485-529 | 2 |
| 4FM5 | B |  |  |  | 474-496, 499-543 | 2 |
| 2ES4 | E | Q05490 |  |  | 246-296, 297-330 | 2 |
| 4IN3 | B | Q05029 |  |  | 489-567, 572-604 | 2 |
| 2VIX | A | Q04640 |  |  | 204-259, 271-306 | 2 |
| 2VJ4 | A |  |  |  | 204-259, 271-305 | 2 |
| 2AW6 | B | Q04114 |  |  | 146-174, 180-224 | 2 |
| 2AWI | G |  |  |  | 180-224, 226-268 | 2 |
| 2AXU | L |  |  |  | 180-224, 226-268 | 2 |
| 2GRL | A |  |  |  | 180-224, 226-268 | 2 |
| 4A7H | C | Q03479 |  |  | 238-288, 295-329 | 2 |
| 3PTK | A | Q01KB2 |  |  | 97-134, 142-179 | 2 |
| 3PTM | B |  |  |  | 97-134, 142-179 | 2 |
| 3PTQ | A |  |  |  | 97-133, 142-179 | 2 |
| 1SV0 | A | Q01842 |  |  | 47-80, 88-119 | 2 |
| 1SV4 | A |  |  |  | 47-80, 88-119 | 2 |
| 4F3V | B | P9WPH9 |  |  | 161-199, 200-249 | 2 |
| 1S56 | A | P9WN25 |  |  | 47-83, 84-129 | 2 |
| 1S61 | A |  |  |  | 47-83, 84-129 | 2 |
| 2GKN | A |  |  |  | 47-83, 84-128 | 2 |
| 2GLN | A |  |  |  | 47-83, 84-129 | 2 |
| 3PNV | A | P9WFV9 |  |  | 421-460, 461-485 | 2 |
| 3AB6 | A | P86383 |  |  | 51-84, 95-117 | 2 |
| 1RRS | A | P83847 |  |  | 72-120, 121-162 | 2 |
| 1TI2 | A | P80563 |  |  | 172-199, 215-252 | 2 |
| 1TI4 | A |  |  |  | 172-199, 215-252 | 2 |
| 1TI6 | A |  |  |  | 172-199, 215-252 | 2 |
| 1VLD | O |  |  |  | 172-199, 215-252 | 2 |
| 1VLE | O |  |  |  | 172-199, 215-252 | 2 |
| 1VLF | O |  |  |  | 172-199, 215-252 | 2 |
| 4KHP | M | P80377 |  |  | 11-40, 41-78 | 2 |
| 4JUW | M |  |  |  | 11-40, 41-68 | 2 |
| 1I94 | M |  |  |  | 11-40, 41-64 | 2 |
| 1PNS | M |  |  |  | 11-40, 41-64 | 2 |
| 1PNX | M |  |  |  | 11-40, 41-64 | 2 |
| 4GKK | M |  |  |  | 11-40, 41-64 | 2 |
| 1HNW | M |  |  |  | 11-40, 41-64 | 2 |
| 1HNX | M |  |  |  | 11-40, 41-64 | 2 |
| 1HNZ | M |  |  |  | 11-40, 41-64 | 2 |
| 1HR0 | M |  |  |  | 11-40, 41-64 | 2 |
| 1IBL | M |  |  |  | 11-40, 41-64 | 2 |
| 1IBM | M |  |  |  | 11-40, 41-64 | 2 |
| 1J5E | M |  |  |  | 11-40, 41-64 | 2 |
| 1N32 | M |  |  |  | 11-40, 41-64 | 2 |
| 1N33 | M |  |  |  | 11-40, 41-63 | 2 |
| 1XMQ | M |  |  |  | 11-40, 41-64 | 2 |
| 1XNQ | M |  |  |  | 11-40, 41-64 | 2 |
| 2F4V | M |  |  |  | 11-40, 41-78 | 2 |
| 2UUA | M |  |  |  | 11-40, 41-68 | 2 |
| 2UUB | M |  |  |  | 11-40, 41-68 | 2 |
| 2UUC | M |  |  |  | 11-40, 41-68 | 2 |
| 2UXB | M |  |  |  | 11-40, 41-63 | 2 |
| 2UXC | M |  |  |  | 11-40, 41-64 | 2 |
| 2UXD | M |  |  |  | 11-40, 41-65 | 2 |
| 2VQE | M |  |  |  | 11-40, 41-63 | 2 |
| 2Y0W | M |  |  |  | 41-73, 74-104 | 2 |
| 2Y0Y | M |  |  |  | 11-40, 41-68 | 2 |
| 2Y10 | M |  |  |  | 41-73, 74-104 | 2 |
| 3T1H | M |  |  |  | 11-40, 41-63 | 2 |
| 4B3M | M |  |  |  | 10-39, 40-63 | 2 |
| 4B3R | M |  |  |  | 10-39, 40-63 | 2 |
| 4B3S | M |  |  |  | 10-39, 40-63 | 2 |
| 4DH9 | M |  |  |  | 11-40, 41-68 | 2 |
| 4DR4 | M |  |  |  | 11-40, 41-64 | 2 |
| 4DR5 | M |  |  |  | 11-40, 41-64 | 2 |
| 4DV6 | M |  |  |  | 11-40, 41-64 | 2 |
| 4G5M | P |  |  |  | 11-40, 41-63 | 2 |
| 4OX9 | M |  |  |  | 11-40, 41-64 | 2 |
| 2ZPA | B | P76562 |  |  | 226-256, 260-290 | 2 |
| 1Y6I | A | P72583 |  |  | 89-127, 136-185 | 2 |
| 3L2O | A | P63208 |  |  | 1093-1129, 1130-1160 | 2 |
| 2JKR | E | P63010 |  |  | 452-474, 487-505 | 2 |
| 2JKT | E |  |  |  | 452-475, 487-518 | 2 |
| 3A2M | B | P59337 |  |  | 131-163, 171-202 | 2 |
| 3A2N | A |  |  |  | 131-173, 178-202 | 2 |
| 1NDW | A | P56658 |  |  | 259-301, 315-352 | 2 |
| 1NDZ | A |  |  |  | 259-301, 315-352 | 2 |
| 1O5R | A |  |  |  | 259-301, 315-352 | 2 |
| 1QXL | A |  |  |  | 259-301, 315-352 | 2 |
| 1V7A | A |  |  |  | 259-301, 315-352 | 2 |
| 1WXY | A |  |  |  | 259-301, 315-352 | 2 |
| 1WXZ | A |  |  |  | 259-301, 315-352 | 2 |
| 2E1W | A |  |  |  | 259-301, 315-352 | 2 |
| 2Z7G | A |  |  |  | 256-298, 312-349 | 2 |
| 1W1I | E |  |  |  | 259-301, 315-355 | 2 |
| 2BGN | G |  |  |  | 259-301, 315-355 | 2 |
| 3GGY | A | P53843 |  |  | 101-150, 162-191 | 2 |
| 1Q1A | A | P53686 |  |  | 33-61, 65-89 | 2 |
| 1NEX | A | P52286 |  |  | 126-158, 159-185 | 2 |
| 3MKS | C |  |  |  | 124-158, 159-186 | 2 |
| 3BG0 | G | P49687 |  |  | 483-520, 522-552 | 2 |
| 3BG1 | C |  |  |  | 483-520, 522-552 | 2 |
| 3IKO | B |  |  |  | 483-516, 520-549 | 2 |
| 1OIZ | B | P49638 |  |  | 11-43, 45-91 | 2 |
| 1D2E | A | P49410 |  |  | 179-211, 216-255 | 2 |
| 1HXJ | B | P49235 |  |  | 52-100, 101-137, 146-186 | 3 |
| 1E55 | B |  |  |  | 57-105, 106-142, 151-191 | 3 |
| 1H49 | B |  |  |  | 57-105, 106-142, 151-191 | 3 |
| 1V08 | A |  |  |  | 57-105, 106-143, 151-191 | 3 |
| 1E1E | A |  |  |  | 106-142, 151-191 | 2 |
| 1E1F | A |  |  |  | 106-142, 151-191 | 2 |
| 1E4L | A |  |  |  | 106-143, 151-191 | 2 |
| 1E4N | A |  |  |  | 106-142, 151-191 | 2 |
| 1E56 | A |  |  |  | 106-143, 151-191 | 2 |
| 1KWP | B | P49137 |  |  | 293-330, 333-375 | 2 |
| 3ML9 | A | P48736 |  |  | 582-633, 634-672 | 2 |
| 3F3F | D | P46673 |  |  | 476-511, 512-549 | 2 |
| 3F3G | C |  |  |  | 476-511, 512-561 | 2 |
| 3F3P | L |  |  |  | 239-267, 268-296 | 2 |
| 4LCT | D | P45432 |  |  | 221-252, 262-294 | 2 |
| 2Y9Y | B | P43596 |  |  | 427-456, 457-503 | 2 |
| 1CXF | A | P43379 |  |  | 227-263, 279-328 | 2 |
| 1D3C | A |  |  |  | 227-263, 279-328 | 2 |
| 2DIJ | A |  |  |  | 227-263, 279-328 | 2 |
| 2O8C | A | P43246 |  |  | 332-378, 383-426 | 2 |
| 2O8D | A |  |  |  | 332-370, 383-435 | 2 |
| 2M2U | A | P42494 |  |  | 104-146, 152-174 | 2 |
| 3VIB | B | P39897 |  |  | 99-154, 155-210 | 2 |
| 3MZL | D | P38968 |  |  | 527-554, 555-585 | 2 |
| 3QXQ | A | P37651 |  |  | 181-226, 239-289 | 2 |
| 1N4D | A | P37028 |  |  | 60-86, 87-143 | 2 |
| 1EK1 | B | P34914 |  |  | 20-45, 46-78 | 2 |
| 3VKG | A | P34036 |  |  | 2200-2225, 2230-2277 | 2 |
| 3VKH | B |  |  |  | 3598-3631, 3632-3652 | 2 |
| 3U33 | E | P33224 |  |  | 115-144, 155-188 | 2 |
| 3IM1 | A | P32639 |  |  | 1989-2012, 2013-2032 | 2 |
| 2RGK | A | P32140 |  |  | 46-90, 106-161, 167-213 | 3 |
| 2RI8 | A | P31723 |  |  | 1263-1308, 1325-1381 | 2 |
| 2RI9 | B |  |  |  | 2263-2308, 2325-2382 | 2 |
| 1KKT | A |  |  |  | 263-308, 325-381 | 2 |
| 1KRE | A |  |  |  | 263-308, 325-381 | 2 |
| 1KRF | A |  |  |  | 263-308, 325-381 | 2 |
| 4HB0 | C | P30822 |  |  | 585-634, 635-686 | 2 |
| 1DWA | M | P29736 |  |  | 105-142, 150-186 | 2 |
| 1DWF | M |  |  |  | 105-142, 150-186 | 2 |
| 1DWG | M |  |  |  | 105-142, 150-186 | 2 |
| 1DWH | M |  |  |  | 105-142, 150-186 | 2 |
| 1DWI | M |  |  |  | 105-142, 150-186 | 2 |
| 1E4M | M |  |  |  | 105-142, 150-186 | 2 |
| 1E6Q | M |  |  |  | 105-142, 150-186 | 2 |
| 1E6S | M |  |  |  | 105-142, 150-186 | 2 |
| 1E6X | M |  |  |  | 105-142, 150-186 | 2 |
| 1E70 | M |  |  |  | 105-142, 150-186 | 2 |
| 1E71 | M |  |  |  | 105-142, 150-186 | 2 |
| 1E72 | M |  |  |  | 105-142, 150-186 | 2 |
| 1E73 | M |  |  |  | 105-142, 150-186 | 2 |
| 1MYR | A |  |  |  | 105-142, 150-186 | 2 |
| 1W9B | M |  |  |  | 105-142, 150-186 | 2 |
| 1W9D | M |  |  |  | 105-142, 150-186 | 2 |
| 2WXD | M |  |  |  | 105-142, 150-186 | 2 |
| 1FDR | A | P28861 |  |  | 142-177, 181-205 | 2 |
| 2XNJ | A |  |  |  | 151-183, 190-214 | 2 |
| 4IZA | A | P28482 |  |  | 245-279, 280-315 | 2 |
| 2JAC | A | P25373 |  |  | 49-81, 84-108 | 2 |
| 1KWO | A | P24733 |  |  | 320-374, 375-406 | 2 |
| 1KK7 | A |  |  |  | 320-364, 373-405 | 2 |
| 1KK8 | A |  |  |  | 320-365, 374-404 | 2 |
| 2AKE | A | P23381 |  |  | 193-230, 233-271 | 2 |
| 2DR2 | A |  |  |  | 193-230, 233-271 | 2 |
| 1W63 | A | P22892 |  |  | 147-184, 185-238 | 2 |
| 3HIL | A | P21709 |  |  | 904-935, 936-964 | 2 |
| 3KKA | A |  |  |  | 914-945, 946-974 | 2 |
| 1FPM | A | P21164 |  |  | 200-232, 234-265 | 2 |
| 4JIM | B |  |  |  | 200-232, 234-265 | 2 |
| 4JKI | A |  |  |  | 200-232, 234-265 | 2 |
| 2R0Q | C | P20384 |  |  | 92-157, 174-200 | 2 |
| 1TAU | A | P19821 |  |  | 610-648, 652-684 | 2 |
| 2VGL | A | P18484 |  |  | 379-419, 435-474 | 2 |
| 1TR2 | A | P18206 |  |  | 218-288, 290-342 | 2 |
| 3AQ6 | B | P17724 |  |  | 39-75, 76-121 | 2 |
| 1FP3 | A | P17560 |  |  | 117-171, 175-231 | 2 |
| 4HOM | A | P15145 |  |  | 849-889, 891-924 | 2 |
| 2QSH | A | P14736 |  |  | 372-392, 393-446 | 2 |
| 2JF4 | A | P13482 |  |  | 154-201, 210-247 | 2 |
| 2JG0 | A |  |  |  | 154-204, 206-255 | 2 |
| 2JJB | A |  |  |  | 154-204, 206-255 | 2 |
| 2WYN | A |  |  |  | 154-204, 206-255 | 2 |
| 2RKJ | A | P12063 |  |  | 352-381, 383-416 | 2 |
| 2HXX | A | P11540 |  |  | 3-35, 51-83 | 2 |
| 2JL9 | A | P11124 |  |  | 295-318, 324-361 | 2 |
| 2HNH | A | P10443 |  |  | 775-821, 831-868, 872-904 | 3 |
| 2HQA | A |  |  |  | 778-821, 831-857, 872-911 | 3 |
| 4JOM | A |  |  |  | 831-860, 872-911 | 2 |
| 1S1H | M | P0CX55 |  |  | 24-53, 54-88 | 2 |
| 2BVN | A | P0CE48 |  |  | 133-165, 170-207 | 2 |
| 4A97 | C | P0C7B7 |  |  | 224-288, 293-317 | 2 |
| 4A98 | A |  |  |  | 224-288, 293-317 | 2 |
| 2VL0 | A |  |  |  | 223-287, 292-315 | 2 |
| 1L2A | F | P0C2S5 |  |  | 82-125, 126-158 | 2 |
| 3LPL | A | P0AFG9 |  |  | 315-350, 358-393 | 2 |
| 3LQ2 | B |  |  |  | 315-350, 358-393 | 2 |
| 2G25 | B | P0AFG8 |  |  | 315-350, 358-391, 402-431 | 3 |
| 1L8A | A |  |  |  | 315-350, 358-393 | 2 |
| 1RP7 | B |  |  |  | 315-350, 358-391 | 2 |
| 2G67 | B |  |  |  | 315-350, 358-393 | 2 |
| 2IEA | B |  |  |  | 315-350, 358-393 | 2 |
| 2QTA | A |  |  |  | 315-350, 358-393 | 2 |
| 2QTC | B |  |  |  | 315-350, 358-393 | 2 |
| 2WC2 | A | P0ACJ8 |  |  | 130-160, 165-200 | 2 |
| 1Q05 | A | P0A9G4 |  |  | 1-34, 37-74 | 2 |
| 1Q06 | A |  |  |  | 1-34, 37-74 | 2 |
| 1Q07 | A |  |  |  | 1-34, 37-74 | 2 |
| 1MJ2 | C | P0A8U6 |  |  | 49-81, 82-104 | 2 |
| 3E1C | O | P0A7V3 |  |  | 68-99, 104-126 | 2 |
| 3DF1 | M | P0A7S9 |  |  | 10-39, 40-61 | 2 |
| 3DF3 | M |  |  |  | 10-39, 40-61 | 2 |
| 3IZW | Q |  |  |  | 10-39, 40-60 | 2 |
| 3KC4 | M |  |  |  | 10-39, 40-61 | 2 |
| 3CP2 | A | P0A6U3 |  |  | 440-497, 505-534 | 2 |
| 3CES | A |  |  |  | 441-488, 505-534 | 2 |
| 1GLA | G | P0A6F3 |  |  | 408-434, 435-462 | 2 |
| 4FWB | A | P0A3G2 |  |  | 134-155, 163-195 | 2 |
| 3FBW | A |  |  |  | 134-155, 163-195 | 2 |
| 4A5P | A | P0A1I5 |  |  | 490-522, 523-560 | 2 |
| 1NUB | A | P09486 |  |  | 228-259, 262-286 | 2 |
| 1BMO | A |  |  |  | 228-259, 262-286 | 2 |
| 2FJR | A | P08707 |  |  | 22-47, 48-85 | 2 |
| 3NXU | B | P08684 |  |  | 168-212, 214-240 | 2 |
| 4K9W | A |  |  |  | 168-212, 214-240 | 2 |
| 1W36 | B | P08394 |  |  | 382-403, 417-447 | 2 |
| 3K70 | B |  |  |  | 382-403, 417-448 | 2 |
| 3BTP | A | P08062 |  |  | 367-402, 405-439 | 2 |
| 1E6Y | B | P07955 |  |  | 2190-2227, 2242-2287,  2293-2347 | 3 |
| 1TNT | A | P07636 |  |  | 3-41, 46-73 | 2 |
| 3FKS | J | P07251 |  |  | 444-477, 480-509 | 2 |
| 3OE7 | J |  |  |  | 444-477, 480-508 | 2 |
| 1QYR | B | P06992 |  |  | 221-244, 245-268 | 2 |
| 1ZQX | A | P06766 |  |  | 260-303, 312-335 | 2 |
| 3UXO | A |  |  |  | 52-80, 89-128 | 2 |
| 1ZQE | A |  |  |  | 52-81, 88-130 | 2 |
| 7ICN | A |  |  |  | 52-81, 88-130 | 2 |
| 8ICW | A |  |  |  | 52-80, 88-120 | 2 |
| 8ICZ | A |  |  |  | 52-81, 88-118 | 2 |
| 9ICP | A |  |  |  | 52-81, 88-130 | 2 |
| 9ICT | A |  |  |  | 52-81, 88-130 | 2 |
| 2QLL | A | P06737 |  |  | 724-750, 758-807 | 2 |
| 3GLF | D | P06710 |  |  | 91-117, 124-160 | 2 |
| 3GLG | D |  |  |  | 91-117, 124-158 | 2 |
| 2W93 | A | P06169 |  |  | 216-252, 261-288 | 2 |
| 3N8X | B | P05979 |  |  | 474-496, 499-543 | 2 |
| 4O1Z | B |  |  |  | 474-496, 499-543 | 2 |
| 2VBJ | A | P05725 |  |  | 77-117, 118-152 | 2 |
| 1MOW | G |  |  |  | 1185-1210, 1211-1250 | 2 |
| 1A4L | A | P03958 |  |  | 259-301, 315-352 | 2 |
| 1A4M | A |  |  |  | 259-301, 315-352 | 2 |
| 3MVI | A |  |  |  | 259-301, 315-352 | 2 |
| 3MVT | A |  |  |  | 259-301, 315-352 | 2 |
| 3KM8 | A |  |  |  | 259-301, 315-352 | 2 |
| 2WYY | F | P03521 |  |  | 195-221, 230-263 | 2 |
| 2H1L | I | P03070 |  |  | 266-298, 299-329 | 2 |
| 2IS2 | B | P03018 |  |  | 218-250, 251-284 | 2 |
| 1KW2 | B | P02774 |  |  | 104-133, 148-193 | 2 |
| 3B9M | A | P02768 |  |  | 271-304, 319-372 | 2 |
| 3JQZ | B |  |  |  | 272-306, 322-372 | 2 |
| 4L9K | A |  |  |  | 274-304, 319-372 | 2 |
| 4LB9 | A |  |  |  | 273-305, 319-372 | 2 |
| 4F4O | J | P01965 |  |  | 25-50, 65-85 | 2 |
| 1MAW | D | P00953 |  |  | 39-73, 76-107 | 2 |
| 2HLD | E | P00830 |  |  | 415-449, 450-475 | 2 |
| 3OEH | X |  |  |  | 274-300, 309-328 | 2 |
| 3OFN | W |  |  |  | 415-449, 450-475 | 2 |
| 2JIZ | E | P00829 |  |  | 415-449, 450-474 | 2 |
| 3IAR | A | P00813 |  |  | 259-301, 315-364 | 2 |
| 1SKW | A | P00581 |  |  | 491-513, 514-541 | 2 |
| 1SL1 | A |  |  |  | 491-513, 514-541 | 2 |
| 3TYK | A | P00557 |  |  | 220-255, 256-302 | 2 |
| 1DE0 | A | P00459 |  |  | 223-251, 257-287 | 2 |
| 2EIK | E | P00426 |  |  | 5-40, 41-77 | 2 |
| 2EIM | E |  |  |  | 5-40, 41-77 | 2 |
| 3ABK | R |  |  |  | 5-40, 41-77 | 2 |
| 3ABL | E |  |  |  | 6-40, 41-77 | 2 |
| 3AG2 | E |  |  |  | 5-40, 41-77 | 2 |
| 3AG3 | E |  |  |  | 5-40, 41-77 | 2 |
| 3AG4 | E |  |  |  | 5-40, 41-77 | 2 |
| 3ASO | E |  |  |  | 5-40, 41-77 | 2 |
| 2Y69 | R |  |  |  | 6-40, 41-77 | 2 |
| 2DKX | A | O95238 |  |  | 20-50, 61-95 | 2 |
| 3Q7J | A | O93655 |  |  | 654-679, 680-718, 719-751 | 3 |
| 1Z5H | B |  |  |  | 680-718, 719-751 | 2 |
| 3SDU | A | O81086 |  |  | 230-252, 256-295 | 2 |
| 4K03 | A | O77059 |  |  | 357-392, 393-436 | 2 |
| 2ZJ2 | A | O73946 |  |  | 437-474, 477-523 | 2 |
| 2ZJ8 | A |  |  |  | 437-473, 477-523 | 2 |
| 2ZJA | A |  |  |  | 437-473, 477-523 | 2 |
| 3WFS | D | O67911 |  |  | 172-211, 216-261 | 2 |
| 4HOD | A | O67854 |  |  | 257-295, 304-335 | 2 |
| 2EH3 | A | O67157 |  |  | 69-126, 127-172 | 2 |
| 2V8P | A | O67060 |  |  | 167-200, 203-237 | 2 |
| 2EYU | A | O66950 |  |  | 296-323, 325-353 | 2 |
| 3H0M | W | O66766 |  |  | 326-357, 371-398 | 2 |
| 1VFG | A | O66728 |  |  | 270-310, 318-351 | 2 |
| 3O0A | A | O66680 |  |  | 241-269, 283-326 | 2 |
| 3R8F | A | O66659 |  |  | 307-327, 329-362 | 2 |
| 4IZM | B | O59010 |  |  | 200-255, 264-301 | 2 |
| 1VFF | A | O58104 |  |  | 112-145, 152-199 | 2 |
| 3FY4 | A | O48652 |  |  | 343-378, 379-417 | 2 |
| 3PPQ | A | O32243 |  |  | 245-265, 267-303 | 2 |
| 1UX8 | A | O31607 |  |  | 38-78, 79-123 | 2 |
| 3USY | A | O25119 |  |  | 204-233, 244-277 | 2 |
| 1N39 | A | O15527 |  |  | 102-126, 133-174 | 2 |
| 1N3C | A |  |  |  | 102-126, 133-174 | 2 |
| 1HU0 | A |  |  |  | 102-126, 133-174 | 2 |
| 1Z6T | A | O14727 |  |  | 317-354, 357-390 | 2 |
| 3U5G | J | O13516 |  |  | 105-143, 159-186 | 2 |
| 4GAX | A | M4GGS1 |  |  | 99-129, 136-181 | 2 |
| 4FJQ | A | M4GGS0 |  |  | 99-129, 136-181, 193-218 | 3 |
| 4GRG | A | L7MTK7 |  |  | 46-79, 80-112, 114-135 | 3 |
| 4ME2 | A | I1HB13 |  |  | 78-113, 114-159, 164-199,  200-237 | 4 |
| 4N2Q | A |  |  |  | 164-197, 200-241 | 2 |
| 4N2S | A |  |  |  | 164-199, 200-240 | 2 |
| 3ZHE | A | G5ECF1 |  |  | 307-345, 347-391 | 2 |
| 4GPK | F | G5DDY8 |  |  | 68-104, 105-140, 301-340,  341-375 | 4 |
| 3B7H | A | F9UL97 |  |  | 19-44, 46-75 | 2 |
| 3WKF | A | F8WRK9 |  |  | 57-113, 119-175 | 2 |
| 3WKI | A |  |  |  | 57-113, 119-175 | 2 |
| 3N11 | A | D0VV09 |  |  | 281-320, 331-355 | 2 |
| 3N12 | A |  |  |  | 281-320, 331-360 | 2 |
| 3N13 | A |  |  |  | 281-320, 331-356 | 2 |
| 3N15 | A |  |  |  | 281-320, 331-356 | 2 |
| 3N17 | A |  |  |  | 281-320, 331-356 | 2 |
| 3N18 | A |  |  |  | 281-320, 331-356 | 2 |
| 3N1A | A |  |  |  | 281-320, 331-356 | 2 |
| 3AQK | A | C9QS13 |  |  | 326-375, 381-430 | 2 |
| 3AQL | A |  |  |  | 326-374, 381-427 | 2 |
| 3AQM | A |  |  |  | 326-375, 381-427 | 2 |
| 3AQN | A |  |  |  | 326-374, 381-418 | 2 |
| 3TJ0 | B | C4LQ26 |  |  | 171-223, 231-269 | 2 |
| 3W53 | A | B9V8P5 |  |  | 88-126, 133-170 | 2 |
| 4M59 | A | B8Y6I0 |  |  | 170-202, 205-239,  521-555, 557-591 | 4 |
| 4DYT | C | B4URF1 |  |  | 109-162, 174-204 | 2 |
| 3UGM | A | B2SU53 |  |  | 321-355, 356-399,  423-456, 457-490 | 4 |
| 3GT5 | A | B2I5L9 |  |  | 115-170, 175-229 | 2 |
| 3LOV | A | B1YKJ9 |  |  | 108-144, 155-206 | 2 |
| 4AYO | A | B0SWV2 |  |  | 185-233, 245-297, 305-352 | 3 |
| 4AYP | A |  |  |  | 185-233, 245-297, 305-352 | 3 |
| 4AYQ | A |  |  |  | 245-297, 305-352 | 2 |
| 4AYR | A |  |  |  | 245-297, 305-352 | 2 |
| 2J5C | B | A6XH05 |  |  | 101-139, 143-176, 184-222 | 3 |
| 3RJV | A | A6TH02 |  |  | 81-115, 117-157, 158-185,  196-227 | 4 |
| 4GML | C | A5YKK6 |  |  | 1242-1273, 1274-1305 | 2 |
| 3BAN | A | A4VVI4 |  |  | 254-294, 300-336 | 2 |
| 3BDK | A |  |  |  | 254-294, 300-336 | 2 |
| 3FVM | A |  |  |  | 254-294, 300-336 | 2 |
| 2K53 | A | A3DK08 |  |  | 5-32, 37-70 | 2 |
| 4O2D | A | A0QWN3 |  |  | 316-351, 355-377 | 2 |
| 2BJC | A | P03023 |  |  | 2-28, 29-61 | 2 |
| 2R63 | A | P16117 |  |  | 13-40, 41-63 | 2 |
| 4GMR | A |  |  |  | 2-35, 36-68, 69-101,  102-135, 136-165 | 5 |
| 4GPM | A |  |  |  | 2-35, 36-68, 69-101,  102-135, 136-158 | 5 |
| 4HB5 | A |  |  |  | 2-35, 36-68, 69-101,  102-135, 136-158 | 5 |
| 4HQD | B |  |  |  | 2-35, 36-68, 69-102,  103-135, 136-159 | 5 |
| 4OSI | A |  |  |  | 389-422, 423-456,  457-505, 627-660, 661-694 | 5 |
| 4OSJ | A |  |  |  | 389-422, 423-456,  457-490, 491-524, 525-572 | 5 |
| 4OTO | B |  |  |  | 287-320, 321-354,  423-456, 457-490, 491-539 | 5 |
| 4OSW | B |  |  |  | 321-354, 355-403,  457-490, 491-539 | 4 |
| 3V6T | B |  |  |  | 389-423, 424-456, 457-490 | 3 |
| 4OSK | A |  |  |  | 457-490, 491-524, 525-574 | 3 |
| 4OSZ | A |  |  |  | 321-354, 355-388, 389-437 | 3 |
| 3BD1 | C |  |  |  | 10-35, 36-62 | 2 |
| 3WG7 | R |  |  |  | 5-40, 41-77 | 2 |
| 1VWX | f |  |  |  | 185-220, 225-250 | 2 |
| 1VWZ | f |  |  |  | 185-220, 225-250 | 2 |
| 1VX1 | f |  |  |  | 185-220, 225-250 | 2 |
| 1VX4 | f |  |  |  | 185-220, 225-250 | 2 |
| 4Q28 | D |  |  |  | 1667-1704, 1717-1753 | 2 |
| 1INZ | A |  |  |  | 35-65, 67-114 | 2 |
| 2OU3 | B |  |  |  | 21-74, 88-126 | 2 |
| 2GRM | A |  |  |  | 180-224, 226-268 | 2 |
| 3WPM | B |  |  |  | 359-383, 384-405 | 2 |
| 3E4B | A |  |  |  | 334-368, 370-403 | 2 |
| 2UVB | A |  |  |  | 1173-1215, 1225-1267 | 2 |
| 1XMO | M |  |  |  | 11-40, 41-64 | 2 |
| 4KVB | M |  |  |  | 11-40, 41-63 | 2 |
| 4NXM | M |  |  |  | 11-40, 41-64 | 2 |
| 4OSH | B |  |  |  | 491-524, 525-572 | 2 |
| 4OSL | B |  |  |  | 423-456, 457-490 | 2 |
| 4OSM | A |  |  |  | 491-524, 525-573 | 2 |
| 4OSQ | B |  |  |  | 423-456, 457-490 | 2 |
| 4OSR | A |  |  |  | 491-524, 525-574 | 2 |
| 4OSS | A |  |  |  | 491-524, 525-573 | 2 |
| 4OST | B |  |  |  | 423-456, 457-490 | 2 |
| 4OSV | A |  |  |  | 491-524, 525-572 | 2 |
| 4OT0 | B |  |  |  | 423-456, 457-490 | 2 |
| 4OT3 | B |  |  |  | 423-456, 457-490 | 2 |

* Predicted ANK repeat region and UniProt repeat annotation are in distinct regions of the protein structure.
