# Supplementary material for: Identifying tandem Ankyrin repeats in protein structures
Source: BMC Bioinformatics. 2014 Dec 30;15(1):6599. doi: 10.1186/s12859-014-0440-9 (PMC4307672; doi:10.1186/s12859-014-0440-9)
Supplement: Additional file 3 — Prediction of Ankyrin repeats by the proposed approach is compared with the UniProt annotation for a set of 30 modelled protein structures. [file 12859_2014_440_MOESM3_ESM.docx]

**Prediction of Ankyrin repeats by the proposed approach is compared with the UniProt annotation for a set of 30 modelled protein structures.**

| **S.No** | **UniProt** | **UniProt annotation** | **AnkPred** |
| --- | --- | --- | --- |
| 1 | Q91ZA8* | 50-79, 83-112 | 15-49, 50-83, 84-106, |
| 2 | Q5I148* | 56-88, 93-123 | 56-92, 93-127, 128-160 |
| 3 | Q7T2B9 | 1-30, 34-65, 67-98 | 1-35, 36-66, 67-94, |
| 4 | Q5ZJJ9* | 72-101, 105-135, 139-168 | 72-104, 105-138, 139-172, 173-193, |
| 5 | Q1RJ94 | 23-52, 80-109, 113-143 | 80-112, 113-142, |
| 6 | Q80T11 | 31-60, 64-93, 97-126 | 31-63, 64-97, 98-130, |
| 7 | Q8L746* | 261-291, 293-320, 324-353 | 160-191, 192-226, 290-323, 324-357, |
| 8 | Q7Z3H0* | 29-58, 64-93, 98-132 | 1-28, 29-62, 64-98, 99-135, 136-165, |
| 9 | Q2KJD8 | 6-35, 39-67, 72-101, 105-131 | 72-105, 106-131, |
| 10 | Q3SX00 | 11-40, 44-73, 77-103, 107-138 | 11-43, 44-76, 77-106, 107-142, |
| 11 | Q9H765* | 52-81, 85-113, 117-146, 150-179 | 25-46, 52-85, 86-117, 118-150, 151-184, |
| 12 | Q91WK7 | 108-137, 141-170, 174-203, 207-239 | 107-140, 141-172, 173-206, |
| 13 | Q6NXT1* | 109-138, 142-171, 175-204, 208-244 | 106-140, 141-173, 174-208, 224-261, 262-289, |
| 14 | O74205 | 289-318, 322-351, 355-384, 413-440 | 289-322, 323-354, 355-383, |
| 15 | Q9U518* | 398-427, 431-460, 497-526, 530-559 | 399-430, 431-464, 465-496, 497-529, 530-558, 563-588, |
| 16 | Q6S5J6* | 287-316, 320-350, 351-384, 388-419 | 287-319, 320-354, 355-387, 388-428, 429-458, |
| 17 | Q9Z205 | 88-127, 132-161, 165-194, 198-227, 231-260 | 91-125, 132-165, 166-198, 199-231, 232-261, |
| 18 | Q99PE2 | 147-179, 180-212, 213-245, 246-278, 279-312 | 148-179, 180-212, 213-246, 247-279, 280-311, |
| 19 | Q9Z1E3* | 73-103, 110-139, 143-172, 182-211, 216-245 | 73-109, 110-142, 143-181, 182-216, 217-249, 250-281, |
| 20 | Q9CR42* | 152-181, 185-214, 218-247, 251-280, 284-315 | 75-111, 119-151, 152-185, 186-218, 219-251, 252-284, 285-305, |
| 21 | Q99J82 | 2-30, 31-63, 64-96, 97-129, 130-174 | 2-32, 33-65, 66-98, 99-131, 132-160, |
| 22 | P83757* | 220-252, 256-285, 287-316, 350-379, 384-413 | 223-253, 254-285, 286-308, 348-381, 382-417, 418-450, |
| 23 | Q6NLQ8 | 50-79, 83-112, 117-147, 177-206, 220-249 | 18-49, 50-82, 83-117, 177-203, 220-256, |
| 24 | Q8WXK3* | 18-47, 51-80, 84-113, 116-145, 149-178, 181-210 | 21-50, 51-84, 85-115, 116-149, 150-175, 181-214, 215-240, |
| 25 | Q8VD46 | 45-74, 78-107, 110-144, 148-177, 181-210, 214-243 | 45-78, 79-109, 110-148, 149-181, 182-214, 215-249, |
| 26 | Q8WWH4 | 45-74, 78-107, 110-144, 148-177, 181-210, 214-243 | 46-77, 78-116, 119-145, 146-176, 181-214, 215-234, |
| 27 | Q554E7 | 159-188, 193-222, 226-255, 260-289, 293-322, 330-359 | 159-192, 193-225, 226-259, 260-293, 294-329, 330-358, |
| 28 | Q6S5H4* | 138-171, 172-201, 205-234, 238-267, 271-300, 304-333, 337-373 | 139-171, 172-204, 205-237, 238-271, 272-303, 304-337, 338-369, 371-399, 400-426, |
| 29 | Q05921 | 24-53, 58-87, 91-120, 124-153, 167-197, 201-234, 238-268, 272-301, 303-328 | 24-57, 58-90, 91-123, 124-150, 167-201, 202-238, 239-272, 273-299, 300-334, |
| 30 | Q96Q27 | 56-85, 89-119, 123-152, 156-185, 189-218, 222-251, 255-284, 288-317, 320-349, 362-391, 392-421, 428-456 | 58-85, 89-122, 123-155, 156-189, 190-221, 222-254, 255-287, 288-319, 320-358, 392-424, 425-451, |

*Extra copy of repeat identified by proposed approach
